# Predictability of leaf traits with climate and elevation: a case study in Gongga Mountain, China

**DOI:** 10.1093/treephys/tpab003

**Published:** 2021-01-13

**Authors:** Huiying Xu, Han Wang, I Colin Prentice, Sandy P Harrison, Genxu Wang, Xiangyang Sun

**Affiliations:** Ministry of Education Key Laboratory for Earth System Modeling, Department of Earth System Science, Tsinghua University, Shuangqing Road, Haidian District, Beijing 100084, China; Joint Center for Global Change Studies (JCGCS), Shuangqing Road, Haidian District, Beijing 100875, China; Ministry of Education Key Laboratory for Earth System Modeling, Department of Earth System Science, Tsinghua University, Shuangqing Road, Haidian District, Beijing 100084, China; Joint Center for Global Change Studies (JCGCS), Shuangqing Road, Haidian District, Beijing 100875, China; Ministry of Education Key Laboratory for Earth System Modeling, Department of Earth System Science, Tsinghua University, Shuangqing Road, Haidian District, Beijing 100084, China; Department of Life Sciences, Imperial College London, Silwood Park Campus, Buckhurst Road, Ascot SL5 7PY, UK; Department of Biological Sciences, Macquarie University, Balaclava Road, North Ryde, NSW 2109, Australia; Ministry of Education Key Laboratory for Earth System Modeling, Department of Earth System Science, Tsinghua University, Shuangqing Road, Haidian District, Beijing 100084, China; School of Archaeology, Geography and Environmental Sciences (SAGES), University of Reading, Reading Berkshire RG6 6AH, UK; Institute of Mountain Hazards and Environment, Chinese Academy of Sciences, Renmin South Road, Wuhou District, Chengdu, China; State Key Laboratory of Hydraulics and Mountain River Engineering, Sichuan University, Renmin South Road, Wuhou District, Chengdu 610065, China; State Key Laboratory of Hydraulics and Mountain River Engineering, Sichuan University, Renmin South Road, Wuhou District, Chengdu 610065, China

**Keywords:** deciduous LMA prediction, elevation gradients, leaf functional traits, leaf nitrogen prediction, optimality-based models, trait–climate relationships

## Abstract

Leaf mass per area (*M*_a_), nitrogen content per unit leaf area (*N*_area_), maximum carboxylation capacity (*V*_cmax_) and the ratio of leaf-internal to ambient CO_2_ partial pressure (χ) are important traits related to photosynthetic function, and they show systematic variation along climatic and elevational gradients. Separating the effects of air pressure and climate along elevational gradients is challenging due to the covariation of elevation, pressure and climate. However, recently developed models based on optimality theory offer an independent way to predict leaf traits and thus to separate the contributions of different controls. We apply optimality theory to predict variation in leaf traits across 18 sites in the Gongga Mountain region. We show that the models explain 59% of trait variability on average, without site- or region-specific calibration. Temperature, photosynthetically active radiation, vapor pressure deficit, soil moisture and growing season length are all necessary to explain the observed patterns. The direct effect of air pressure is shown to have a relatively minor impact. These findings contribute to a growing body of research indicating that leaf-level traits vary with the physical environment in predictable ways, suggesting a promising direction for the improvement of terrestrial ecosystem models.

## Introduction

A number of leaf traits are diagnostic of photosynthetic processes. The ratio of leaf-internal to external CO_2_ (χ) reflects the stomatal regulation of CO_2_ uptake, which has to be balanced against water loss (Wang et al. 2017*b*). The maintenance of transpiration involves a carbon cost, in the form of respiration by living parenchyma cells, to maintain active water transport tissues. The maximum capacity of carboxylation at a standard temperature of 25 °C, *V*_cmax25_, is a measure of the control of photosynthesis by the amount of the enzyme (Rubisco) responsible for carbon fixation (Wang et al. 2020). The maintenance of photosynthetic capacity also incurs a substantial carbon cost in the form of leaf respiration to support protein synthesis. Leaf mass per unit area (*M*_a_) determines the total carbon cost of leaf construction (Wright et al. 2004). Nitrogen is required for both metabolic processes and leaf construction (Lambers and Poorter 1992, Onoda et al. 2004). Leaf nitrogen content per unit area (*N*_area_) thus provides a combined measure of the metabolic and structural costs.

Empirical analyses of large trait data sets have shown that variation in each of these traits is related to climate, and indeed specific climate variables can be shown to influence individual processes (Wright et al. 2005, Ordoñez et al. 2009, Meng et al. 2015). The *V*_cmax25_ is primarily determined by the amount of Rubisco, while the activity of Rubisco varies with the leaf temperature (Devos et al. 1998, Rokka et al. 2010). Vapor pressure deficit represents the atmospheric moisture demand: it is the difference between the saturated vapor pressure of water (a function of temperature) and the actual vapor pressure, which depends on the atmospheric pressure and moisture content. Vapor pressure deficit influences stomatal behavior and thereby induces variation in χ (Wang et al. 2017*b*). The amount of light reaching the leaves influences *M*_a_ and *N*_area_ within the canopy (Werger and Hirose 1991, Peltoniemi et al. 2012). Both also vary with latitude because this determines total incident radiation and day length (Forsythe et al. 1995). Analyses have shown that the variability in each of these traits is largely independent of variability in the others (Yang et al. 2019).

Elevational transects provide examples of trait variability along environmental gradients (Jian et al. 2009, Asner and Martin 2016, Asner et al. 2017, Pfennigwerth et al. 2017). Although this variability is partly related to the changes in climate with elevation, the impact of changing elevation on air pressure is also thought to be significant (Gale 1972, Terashima et al. 1995, Wang et al. 2014, Wang et al. 2017*a*). Reduction in air pressure at higher elevations lowers the partial pressure of oxygen. All else being equal, it also decreases the water vapor pressure and increases the atmospheric transmissivity to solar radiation. The reduction in partial pressure of oxygen increases the affinity of Rubisco for CO_2_, which reduces photorespiration. The effects of decreasing water vapor pressure and increasing transmissivity are often countered by decreasing temperature and increasing cloudiness. Nonetheless, their contribution (compared with the situation at constant elevation) is to increase the vapor pressure deficit—because atmospheric pressure automatically declines with elevation, while the saturated vapor pressure does not—resulting in higher water transport costs and lower χ, and to increase absorbed light, resulting in increased *V*_cmax25_, *M*_a_ and *N*_area_ (Wang et al. 2017*a*).

It is difficult to disentangle the effects of air pressure and climate along elevation gradients because of their covariation. Attempts to separate out climate and elevation empirically by comparing low-elevation sites at higher latitude with high-elevation sites at lower latitude (Körner et al. 1991) have distinguished the impacts of temperature from air pressure but have not addressed specific climate influences. However, understanding the relative importance of air pressure effects on photosynthesis could be important in the face of projected climate changes, in particular, given the apparent sensitivity of high-elevation sites to these changes (Stocker et al. 2013, Settele et al. 2015).

Recent progress in the application of optimality theory to predict trait variation (Prentice et al. 2014, Dong et al. 2017, Wang et al. 2017*b*) offers an alternative way to examine the impacts of climate and elevation on photosynthesis. Optimality theory is predicated on the idea that through evolutionary processes (including selection for plasticity as well as environmental filtering of lineages) plants are adapted to the environmental conditions under which they live. The values of photosynthetic parameters are then predicted as the result of trade-offs between competing requirements, such as the need to balance CO_2_ uptake against water loss. The balance between maintaining carboxylation capacity and transpiration capacity can be described in terms of the least-cost hypothesis (Wright et al. 2003, Prentice et al. 2014), which states that plants minimize the combined costs of maintaining these capacities. This hypothesis allows us to predict χ. The coordination hypothesis (Chen and Reynolds 1997, Maire et al. 2012, Wang et al. 2017*b*) indicates that carbon gain is maximized through balancing light and Rubisco limitations on photosynthesis. This hypothesis allows us to predict *V*_cmax25_ (Smith et al. 2019). The need to allocate nitrogen to structural and metabolic processes allows us to predict *N*_area_ as a function of *V*_cmax25_ and *M*_a_ (Dong et al. 2017). According to the optimal leaf longevity (LL) hypothesis (Kikuzawa 1991), plants maximize the time-averaged net carbon gain of leaves, taking into account the construction costs (amortized over the leaf lifetime) and the decline in photosynthetic capacity with increasing age. This hypothesis allows *M*_a_ to be predicted from LL. The LL of deciduous species is constrained by growing season length (gsl); thus, *M*_a_ of deciduous species should be predictable fromgsl.

In this study, we draw on these theoretical developments to predict trait variability in response to climate and elevation gradients in the Gongga Mountain region, China. We develop a new optimality model to predict *M*_a_ of deciduous species and a simplified optimality approach to predict *N*_area_. These optimality models were developed independently of the observations used in this study and require no calibration. We show that these models capture observed variations in photosynthetic traits at sites in the Gongga Mountain region. We then use these models to quantify the relative contribution of different factors to the observed changes in trait values at these sites.

## Materials and methods

### Study sites

**Figure 1.**
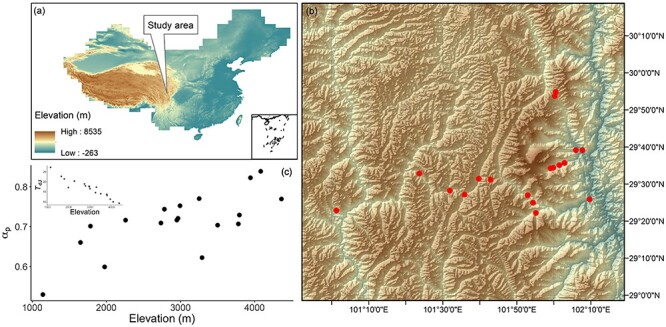
The study area. (a) The location of the Gongga Mountain region in China, (b) spatial distributions of the sampled sites in the Gongga Mountain region, shown by red dots, and (c) the daytime temperature in July (*T*_dJ_) and the ratio of annul actual evapotranspiration to annual potential evapotranspiration (α_p_) at the sampled sites. The background to plots (a) and (b) shows elevation.

We collected photosynthetic trait data from 18 sites in the Gongga Mountain region of Sichuan Province, China (Figure 1a and b). The study area extends from 29° 22′ to 29° 55′ N and from 101° 1′ to 102° 9′ E. The sampled sites span an elevation gradient from 1143 to 4361 m, and as a result, there is a considerable gradient in growing season temperature (see Table S1 available as Supplementary data at *Tree Physiology* online). Sites from the western part of the Gongga Mountain region tend to be drier than the sites at a corresponding elevation in the eastern part, and thus, our data set also samples a large moisture gradient (see Table S1 available as Supplementary data at *Tree Physiology* online). The vegetation at lower elevations is deciduous broad-leaved forest dominated by Betulaceae, Urticaceae, Caprifoliaceae and Rosaceae, and it is replaced by evergreen needle-leaved forest and subsequently by deciduous shrubland dominated by Pinaceae and/or Rosaceae and Ericaceae (see Table S2 available as Supplementary data at *Tree Physiology* online) with increasing elevation. Although evergreen woody species are present at all of the sites (see Table S2 available as Supplementary data at *Tree Physiology* online), and trait measurements were made on these species, our subsequent analyses of photosynthetic traits focused entirely on the deciduous species because of the difficulty of obtaining reliable estimates of leaf age based on a single sampling of a site.

### Sample collection and analysis

Trait data were measured in late July 2018 and early August 2019 during the active growing season in the Gongga Mountain region. We used a stratified sampling strategy at each site in order to sample the dominant species in each canopy stratum. In forest sites, we sampled a minimum of five tree, five shrub and five forb species at each site and also sampled graminoids, lianas and climbers, and pteridophytes when present (see Table S2 available as Supplementary data at *Tree Physiology* online). At the highest elevation sites (3794, 3943, 4081 and 4361 m), where shrubs form the upper canopy, we only sampled shrubs and forbs (and at the highest site one pteridophyte), but again, we sampled a minimum of five species in each category. All samples were taken from the outer canopy. Measurements were made on the young but fully expanded leaves attached to the cut branch.

Our analyses focus on four leaf traits: (i) leaf mass per unit area (*M*_a_, g biomass m^−2^), (ii) the maximum capacity of carboxylation at 25 °C (*V*_cmax25_, μmolC m^−2^ s^−1^), (iii) the ratio of leaf-internal to ambient CO_2_ partial pressure (χ, unitless) and (iv) leaf nitrogen content per unit area (*N*_area_, g m^−2^). (see Table 1 for definitions of parameters and other abbreviations frequently used in the text.) The *M*_a_ was obtained from the measurements of leaf area and dry weight following standard protocols (Cornelissen et al. 2003). Leaf area was taken as the projected area of a leaf, or leaflet for compound leaves, using a LiDE 220 Scanner (Canon Inc., Huntington, NY, USA). Dry weight was obtained after air-drying for several days and then after oven-drying at 75 °C for 48 h to constant weight. The *M*_a_ value of one species at each site was the average of three measurements made on leaves from multiple individuals. Leaf nitrogen content was measured using an Isotope Ratio Mass Spectrometer (Thermo Fisher Scientific Inc., Carlsbad, CA, USA). The *N*_area_ was calculated from *M*_a_ and leaf nitrogen content. Leaf nitrogen content (for *N*_area_) and stable carbon isotope (δ^13^C, for χ) measurements were made on pooled samples of leaves from multiple individuals.

**Table 1:** Parameters and abbreviations frequently used in the text. The table provides information on the meaning and units

Parameters and abbreviations	Unit	Description	
χ	Pa Pa^−1^	The ratio of leaf-internal to ambient CO_2_ partial pressures	
*M*_a_	g biomass m^−2^	Leaf mass per area	
*N*_area_	g m^−2^	Leaf nitrogen content per area	
*V*_cmax_	μmolC m^−2^ s^−1^	The maximum capacity of carboxylation	
*V*_cmax25_	μmolC m^−2^ s^−1^ molC m^−2^ day^−1^	The maximum capacity of carboxylation at standard 25 °C Used when calculating *b* in *M_a_* section	
*T*_g_	°C	Mean temperature during the growing season (mean daily temperature above a baseline of 0 °C)	
*D*_0_	kPa	Mean vapor pressure deficit during the growing season	
*R*_0_	μmol photon m^−2^ s^−1^	Mean photosynthetically active radiation during the growing season	
*R*_LAI_	mol photon m^−2^ day^−1^	Mean leaf-area-index-weighted photosynthetically active radiation during the growing season	
*f*	day day^−1^	The ratio of gsl to the number of days in the year	
MAP	mm	Mean annual precipitation	
α_p_	mm mm^−1^	The ratio of annual actual evapotranspiration to annual potential evapotranspiration	
*T*_dJ_	°C	Mean daytime temperature of July	
Γ^*^	Pa	The photorespiratory compensation point	
*c*_a_	Pa	Ambient CO_2_ partial pressure	
*c*_i_	Pa	Internal CO_2_ partial pressure	
β	unitless	The ratio at 25 °C of the unit costs of maintaining carboxylation and transpiration capacities (estimated as 146)	
*Κ*	Pa	The effective Michaelis–Menten coefficient of Rubisco	
*K*_c_	Pa	The Michaelis–Menten coefficients of Rubisco for carboxylation	
*c*	unitless	A constant proportional to the unit carbon cost for the maintenance of electron transport capacity (0.41)	
LL	day	Leaf longevity	
*b*	day	The potential age when leaves can no longer photosynthesize and assimilate CO_2_	
*k*	g biomass mol C^−1^	Scaling factor	
*I*_abs_	mol photon m^−2^ day^−1^	The photosynthetically active radiation absorbed by leaves	
CC	gC gC^−1^	A constant representing the construction carbon cost per unit leaf mass carbon	
*A*_a_	g biomass m^−2^ day^−1^	Daily carbon assimilation rate per unit leaf area	
φ_0_	μmol C μmol^−1^ photon	The intrinsic quantum efficiency of photosynthesis	
	mol C mol^−1^ photon	Used in Eq. (12) of the *M*_a_ section	
*N*_rubisco_	g m^−2^	Nitrogen content in Rubisco enzymes	
*N*_structure_	g m^−2^	Nitrogen content in leaf structure	

We used a portable infrared gas analyzer system (LI-6400; Li-Cor Inc., Lincoln, NE, USA) to make the leaf gas-exchange measurements. Sunlit terminal branches from the upper canopy were collected and re-cut under water immediately prior to measurement. Measurements were made in the field with relative humidity and chamber block temperature close to that of the ambient air at the time of measurement and with a constant airflow rate (500 μmol s^−1^). The *V*_cmax_ was calculated from the light-saturated rate of net CO_2_ fixation at ambient CO_2_ using the one-point method (De Kauwe et al. 2016) and was adjusted to a standard temperature of 25 °C (*V*_cmax25_) using the method of Bernacchi et al. (2003). The *V*_cmax_ value of one species at each site was obtained from one individual only due to the time-consuming nature of the measurement.

Carbon isotopic values (δ^13^C) were measured using an Isotope Ratio Mass Spectrometer (Thermo Fisher Scientific Inc., Carlsbad, CA, USA). Estimates of χ were made using the simplified method of Ubierna and Farquhar (2014) to calculate isotopic discrimination (Δ) from δ^13^C by considering discrimination during stomatal diffusion, carboxylation and photorespiration, thus following the relationship:

(1) \begin{equation*} \chi =\frac{\Delta +\frac{f^{\prime }{\Gamma}^{\ast }}{c_a} \ \hbox{--} \ {a}_s}{b^{\prime} \ \hbox{--} \ {a}_s}, \end{equation*}

where *a*_s_, *b*′ and *f*′ are the fractionations associated with diffusion in air (4.4‰), Rubisco carboxylation (30‰) and photorespiration (16‰), respectively. Γ^*^ is the photorespiratory compensation point and *c*_a_ is the ambient CO_2_ partial pressure.

### Climate data

In situ climate data were only available for five (1785, 2782, 2993, 3251 and 3943 m) of the 18 sampled sites. We therefore estimated the climate at each site consistently by interpolation between a larger set of weather stations in the region (17 stations, see Figure S1 available as Supplementary data at *Tree Physiology* online) for the period from January 2017 to December 2019 (http://data.cma.cn/data/cdcdetail/dataCode/SURF_CLI_CHN_MUL_MON.html) to create seasonal climatologies of monthly maximum and minimum temperatures, fraction of sunshine hours, water vapor pressure and precipitation. These 17 stations range in elevation from 422 to 3951 m. We then used the elevationally sensitive ANUSPLIN interpolation scheme (Hutchinson and Xu 2004) to provide estimates of these meteorological variables at each of the sites. The monthly estimates at each site were converted to daily values by linear interpolation in order to calculate the bioclimatic variables required as inputs to our models, specifically, growing season mean daytime temperature (*T*_g_), growing season mean vapor pressure deficit (*D*_0_) and growing season mean photosynthetically active radiation (*R*_0_), where the growing season was defined as the period when the daily temperature is above 0 °C. We also calculated the ratio of gsl to the number of days in the year (*f*) and the leaf-area-index-weighted *R*_0_ (*R*_LAI_) to represent the effect of light interception by different layers in the canopy (Dong et al. 2017). The average leaf area index during July and August (i.e., the months the trait data were collected) in 2018 and 2019 was derived from the MODIS leaf area index product (MCD15A3H: https://modis.gsfc.nasa.gov/). An annual moisture index (α_p_, an estimate of the ratio of annual actual evapotranspiration to annual potential evapotranspiration) was calculated from the monthly temperature, precipitation and fraction of sunshine hours at each site using the simple process-led algorithms for simulating habitats (SPLASH) model (Davis et al. 2017). Given the large difference between daytime and night-time temperatures at high elevations, we also calculated the mean daytime temperature of July (*T*_dJ_) by approximating the daily temperature cycle with a sine curve:

(2) \begin{equation*} {T}_{\mathrm{dJ}}={T}_{\mathrm{max}}\left\{\frac{1}{2}+\frac{{\left(1 \ \hbox{--} \ {\mathrm{x}}^2\right)}^{\frac{1}{2}}}{2\,{\mathrm{cos}}^{ \ \hbox{--} \ 1}\textit{x}}\right\}+{T}_{\mathrm{min}}\left\{\frac{1}{2} \ \hbox{--} \ \frac{{\left(1 \ \hbox{--} \ {\mathrm{x}}^2\right)}^{\frac{1}{2}}}{2\,{\mathrm{cos}}^{ \ \hbox{--} \ 1}\textit{x}}\right\}, \end{equation*}

where *T*_max_ is the mean daily maximum air temperature, *T*_min_ is the mean daily minimum air temperature and *x =* − tan φ tan δ, where φ is site latitude and δ is the average solar declination in July.

Comparison of the interpolated bioclimate variables with the values calculated using in situ data at the five sites where such data are available (see Figure S2 available as Supplementary data at *Tree Physiology* online) suggests that the ANUSPLIN interpolation provides robust estimates of the patterns of variation in climate across sites although, except for July temperature, the absolute values differ.

### Trait data analysis

Analyses of the trait data focused on the predominant deciduous component of each community. We used redundancy analysis (RDA: Legendre and Legendre 2012) to determine the main patterns of trait variation using species average values from each site, assess how much of this variation is explained by environmental factors and determine the correlations between traits and environment. The RDA was performed using the *vegan* package in R (Oksanen et al. 2017). In order to compare the trait variability within and across sites, we calculated the coefficient of variation (CV: Brown 1998), a standardized measure of the dispersion of a frequency distribution, for the data set as a whole and at each site, for each of the traits independently.

We used generalized additive models (GAMs) to analyze trait variability with α_p_ and elevation. The GAMs (Hastie and Tibshirani 1990) allow flexible relationships between response and predictor variables to be fitted to the data, avoiding the need to assume the form of the function in advance. Convex hulls were used to exclude areas of the fitted surface that were not well constrained by observations. The GAMs were fitted using the *mgcv* package (Wood 2001), and α-convex hull was produced using *alphahull* package in R (Rodríguez Casal and Pateiro López 2010).

### Trait prediction

We used existing optimality based models of χ and *V*_cmax25_ and new models of *M*_a_ and *N*_area_ to predict the distribution of traits with climate and elevation across the sites. We used gsl as a proxy for the LL of deciduous plants. Specific photosynthetic traits adjust to the environmental conditions over different timeframes (Xu and Baldocchi 2003, Jiang et al. 2020), so we tried two alternative measures of temperature (*T*_g_ and *T*_dJ_) as predictors. The models for χ and *V*_cmax25_ apply for both deciduous and evergreen species.

#### The model for χ

This model is based on the assumption of evolutionary optimality in the trade-off between the costs of transpiration and carbon gain. The least-cost hypothesis predicts that plants minimize the total costs of photosynthesis, i.e., the requirement to maintain capacities for both carboxylation and transpiration (Wright et al. 2003, Prentice et al. 2014). Using the standard photosynthesis model due to Farquhar et al. (1980), Wang et al. (2017*b*) showed that χ could be predicted by:

(3) \begin{equation*} \chi =\frac{\Gamma^{\ast }}{{c}_{\mathrm{a}}}+\frac{\xi \left(1 \ \hbox{--} \ \frac{\Gamma^{\ast }}{{c}_{\mathrm{a}}}\right)}{\xi +\sqrt{D_0}}, \end{equation*}

where

(4) \begin{equation*} \xi =\sqrt{\frac{\beta \left(K+{\Gamma}^{\ast}\right)}{1.6\eta}}, \end{equation*}

and

(5) \begin{equation*} K={K}_{\mathrm{c}}\left(1+\frac{P_{\mathrm{o}}}{K_{\mathrm{o}}}\right). \end{equation*}

Here Γ^*^ is the photorespiratory compensation point, and *c*_a_ is the ambient CO_2_ partial pressure. The η is the viscosity of water relative to its value at 25 °C. The β is the ratio at 25 °C of the unit costs of maintaining carboxylation and transpiration capacities. Based on a global compilation of leaf ^13^C measurements, Wang et al. (2017*b*) estimated β = 146. The *Κ* is the effective Michaelis–Menten coefficient of Rubisco. The *K*_c_ and *K*_o_ are the temperature-dependent Michaelis–Menten coefficients for carboxylation and oxygenation, with reference values at 25 °C of 39.97 Pa and 27.48 kPa, respectively (Bernacchi et al. 2001). The *P*_o_ is the ambient partial pressure of O_2_. The composite variable ξ determines the sensitivity of χ to *D*_0_. This dependence is influenced by temperature (via Γ^*^, *K* and η) and O_2_ pressure (via *K*) according to Eqs (4) and (5).

#### The model for *V*_cmax25_

The coordination hypothesis states that plants coordinate light-limited and Rubisco-limited photosynthesis rates so as to be equal under average daytime conditions (Chen et al. 1993). This coordination ensures that the use of absorbed light is maximized without incurring additional maintenance costs for *V*_cmax_. The *V*_cmax_ acclimated to growth temperature can be predicted from the universal model of carbon uptake proposed by Wang et al. (2017*b*):

(6) \begin{equation*} {V}_{\mathrm{cmax}}={\varphi}_0{R}_0\left(\frac{c_{\mathrm{i}}+K}{c_{\mathrm{i}}+2{\Gamma}^{\ast }}\right)\sqrt{1 \ \hbox{--} \ {\left(\frac{c}{m}\right)}^{\frac{2}{3}}}, \end{equation*}

(7) \begin{equation*} m=\left(\frac{c_{\mathrm{i}} \ \hbox{--} \ {\Gamma}^{\ast }}{c_{\mathrm{i}}+2{\Gamma}^{\ast }}\right), \end{equation*}

where φ_0_ is the intrinsic quantum efficiency of photosynthesis (0.085 μmol C μmol^−1^ photon), and *c*_i_ is the leaf-internal CO_2_ partial pressure, which is the product of observed χ and *c*_a_. The *c* is a constant proportional to the unit carbon cost for the maintenance of electron transport capacity (a value of 0.41 was estimated from an independent global data set on photosynthetic capacities). The *m* represents the effect of subsaturating CO_2_ on the light-limited rate of photosynthesis.

The kinetic response of Rubisco to temperature allows *V*_cmax25_ to be estimated from *V*_cmax_ at growth temperature (*T*_g_), by the following relationship:

(8) \begin{equation*} {V}_{\mathrm{cmax}}={V}_{\mathrm{cmax}25}{f}_{\mathrm{v}}, \end{equation*}

(9) \begin{equation*} {f}_{\mathrm{v}}={\mathrm{e}}^{H_{\mathrm{a}}({T}_{\mathrm{g}} \ \hbox{--} \ 298.15/(298.15{T}_{\mathrm{g}}R))}\times \frac{\left[1+{\mathrm{e}}^{\left(298.15\Delta S \ \hbox{--} \ {H}_{\mathrm{d}}\right)/(298.15R)}\right]}{\left[1+{\mathrm{e}}^{({T}_{\mathrm{g}}\Delta S \ \hbox{--} \ {H}_{\mathrm{d}})/({T}_{\mathrm{g}}R)}\right]}, \end{equation*}

where *H*_a_ is the activation energy (71,513 J mol^−1^), *R* is the universal gas constant (8.314 J mol^−1^ K^−1^), *H*_d_ is the deactivation energy (200,000 J mol^−1^) and ∆*S* is an entropy term (J mol^−1^ K^−1^) calculated using a linear relationship with *T*_g_, with a slope of 1.07 J mol^−1^ K^−2^ and intercept of 668.39 J mol^−1^ K^−1^ (Kattge and Knorr 2007).

#### A new model for *M*_a_

The *M*_a_ contributes to determining how much leaf area can be displayed for a given amount of carbon allocated to above-ground tissues (Cui et al. 2019). There is a universal trade-off between *M*_a_ and LL across growth forms, plant functional types (PFTs) and biomes, known as the ‘leaf economics spectrum’ (Wright et al. 2004). The spectrum runs from a ‘fast’ to a ‘slow’ economic strategy. Plants adopting a fast economic strategy have rapid returns on investment (low *M*_a_) and short longevity (low LL), while plants adopting the slow strategy have high *M*_a_ and high LL.

Here we propose a novel model for *M*_a_, which combines three optimality-based predictions. We start from the model proposed by Kikuzawa (1991). By assuming that the average net carbon gain by a leaf during its lifetime is maximized, this model provides an optimality-based prediction of the trade-off between *M*_a_ and LL:

(10) \begin{equation*} \mathrm{LL}=\sqrt{\frac{2b\ast \mathrm{CC}\ast{M}_{\mathrm{a}}}{A_{\mathrm{a}}}}. \end{equation*}

Here *b* (day) is the potential age at which leaves can no longer photosynthesize, CC (gC gC^−1^) is the construction cost per unit mass of leaf carbon and *A*_a_ (g biomass m^−2^ day^−1^) is the daily carbon assimilation rate per unit leaf area. The *M*_a_ can be written as a function of LL, *b* and *A*_a_ from Eq. (10). Consequently, understanding the environmental responses of these three traits is the key to predicting *M*_a_.

Second, Xu et al. (2017) showed that *b* is approximately proportional to *M*_a_ and inversely proportional to *V*_cmax25_:

(11) \begin{equation*} b=\frac{u\ {M}_{\mathrm{a}}}{k\ {V}_{\mathrm{cmax}25}}. \end{equation*}

Here *u* ≈ 8889 (dimensionless), estimated from a meta-analysis of data on 49 species across temperate and tropical biomes (Xu et al. 2017), and *k* is a scaling factor (30 g biomass mol C^−1^).

Third, the coordination hypothesis allows optimal values of *V*_cmax_ to be predicted by equating the Rubisco-limited assimilation rate with the electron transport limited rate under typical daytime conditions that include temperature, vapor pressure deficit, ambient CO_2_ and the photosynthetically active radiation absorbed by leaves (*I*_abs_). The model has the mathematical form of a ‘light--use efficiency model’: that is, modeled total photosynthesis over any period is proportional to the total light absorbed during that period, which is consistent with classical studies on crop growth (Wang et al. 2017*b*). For this derivation, we made the simplifying assumption that the maximum rate of electron transport (*J*_max_) is large enough that the square-root term in Eq. (6) can be neglected. We substituted Eqs (8) and (9) into (11) to predict *b* from *M*_a_ and *V*_cmax_, which is then predictable from φ_0_, *I*_abs_, *c*_i_, Γ^*^ and *K*. In this way, we obtained a theoretical prediction of *M*_a_:

(12) \begin{equation*} {M}_{\mathrm{a}}={\varphi}_0{I}_{\mathrm{a}\mathrm{bs}}\ \mathrm{LL}\ k\sqrt{\frac{\left({c}_{\mathrm{i}} \ \hbox{--} \ {\Gamma}^{\ast}\right)\left({c}_{\mathrm{i}}+K\right)}{\left(2u\mathrm{CC}{f}_{\mathrm{v}}\right){\left({c}_{\mathrm{i}}+2{\Gamma}^{\ast}\right)}^2}}. \end{equation*}

In addition to the implied proportionality of *M*_a_ with both absorbed light and LL, Eq. (12) indicates the existence of a composite temperature effect due to the temperature dependencies of χ, Γ^*^, *K* and *f*_v_. In order to separate these dependencies, estimate the net effect of temperature more easily and account for the moisture effect detected in the China Plant Trait database (Wang et al. 2018), we obtained the partial derivative of ln(*M*_a_) in Eq. (12) with respect to temperature (*T*_g_) and evaluated the result under standard environmental conditions. This predicts a decline in ln(*M*_a_), for a given LL and *I*_abs_, of ≈3% per degree increase in growth temperature (*T*_g_). In addition, all the constants (φ_0_, *u, k*, CC and reference values of *f*_v_, *K*, *c*_i_ and Γ^*^ at 25 °C) are combined into a single parameter *C*_1_ to reduce the complexity of the model. A linearized equation for predicted *M_a_* can then be derived as:

(13) \begin{equation*} \ln \left({M}_{\mathrm{a}}\right)=\ln \left({I}_{\mathrm{a}\mathrm{bs}}\right) \ \hbox{--} \ 0.03\ {T}_{\mathrm{g}}+\ln \left(\mathrm{LL}\right)+\ln \left({C}_1\right), \end{equation*}

where *C*_1_ is a free parameter. For deciduous species, there is an additional constraint on LL by gsl in Eq. (13), thus we obtained the equation for deciduous species:

(14) \begin{equation*} \ln \left({M}_{\mathrm{a}}\right)=\ln \left({I}_{\mathrm{a}\mathrm{bs}}\right) \ \hbox{--} \ 0.03\ {T}_{\mathrm{g}}+\ln (f)+\ln \left({C}_2\right), \end{equation*}

where *f* is the ratio of gsl to the number of days in the year. Thus, information on the number of days in a year is considered in the free parameter (ln(*C*_2_) = ln(*C*_1_) + ln(365)), resulting in changing of *C*_1_ to *C*_2_. The *C*_1_ and *C*_2_ are unknown a priori but could be estimated from observations.

Although not included in this theoretical derivation, a strong negative effect of increasing moisture availability on *M*_a_ has been reported (Meng et al. 2015). We used the ratio of actual to potential evapotranspiration (α_p_) as an index of moisture availability in order to estimate this effect from the data. Thus, parameter *C*_2_ is further replaced by *C*_3_ to denote the parameter difference in Eq. (14) and Eq. (15) after the moisture effect is included.

We used an independent data set of ln(*M*_a_) for 621 deciduous species from the China Plant Trait database (Wang et al. 2018) to estimate the parameter *C*_3_. Using *R*_LAI_ to represent the averaged light absorbed by leaves, we regressed the observations of ln(*M*_a_) against ln(*R*_LAI_), *T*_g_, ln(*f*) and ln(α_p_) and obtained an estimate of ln(*C*_3_) of 1.70. The predictors in this analysis explained 53% of the variation in *M*_a_, and the fitted slopes of *R*_LAI_, *T*_g_ and ln(*f*) were quantitatively consistent with their theoretical values as given in Eq. (14). Thus, the final model for *M*_a_ was:

(15) \begin{eqnarray*} \ln \left({M}_{\mathrm{a}}\right)\!\!\!\!\!\!\!\!\!\!&&=1.22\ln \left({R}_{\mathrm{LAI}}\right)+0.78\ln (f) \ \hbox{--} \ 0.06\ {T}_{\mathrm{g}} \ \hbox{--} \ 0.60\ \ln \left(\alpha \mathrm{p}\right)\nonumber\\ &&\quad+\,1.70. \end{eqnarray*}

#### A simple model for *N*_area_

The *N*_area_ represents the sum of nitrogen in both metabolic and structural components of a leaf. Dong et al. (2017) proposed a model to predict *N*_area_ from *M*_a_ and *V*_cmax25_ by assuming (based on previously published analyses) that (i) *V*_cmax25_ is proportional to nitrogen in Rubisco and (ii) non-photosynthetic nitrogen is almost proportional to *M*_a_. The model of Dong et al. (2017) is as follows:

(16) \begin{equation*} {N}_{\mathrm{area}}=9.5{N}_{\mathrm{rubisco}}+{N}_{\mathrm{structure}}, \end{equation*}

(17) \begin{equation*} {N}_{\mathrm{structure}}={10}^{ \ \hbox{--} \ 2.67}{M_{\mathrm{a}}}^{0.99} \end{equation*}

and

(18) \begin{equation*} {N}_{\mathrm{rubisco}}=0.003135{V}_{\mathrm{cmax}25}. \end{equation*}

The coefficient of *N*_rubisco_ in Eq. (16) reflects the allocation of total metabolic nitrogen to Rubisco, which however, varies among species. We used the observed *M*_a_ and *V*_cmax25_ in this study to estimate *N*_structure_ and *N*_rubisco_ in Eqs (17) and (18), then fitted a regression of metabolic nitrogen (estimated as the difference between *N*_area_ and *N*_structure_) against *N*_rubisco_ to estimate this coefficient for the deciduous species from the Gongga sites. We obtained a value for the coefficient of *N*_rubisco_ of 7.2, which is within the predicted range given in Dong et al. (2017).

However, there is considerable uncertainty in Eq. (18), which describes the maximal catalytic turnover rate of Rubisco at 25 °C (von Caemmerer et al. 1994, Harrison et al. 2009) as well as in Eqs (16) and (17). To simplify the calculations and avoid these uncertainties, we adopted an alternative method to estimate *N*_area_ directly by regression as a linear combination of all observed *M*_a_ and *V*_cmax25_ (without intercept) in this study, yielding a simpler model that applies to non-nitrogen-fixing plants:

(19) \begin{equation*} N\mathrm{_{area}}=0.02M\mathrm{_{a}}+0.003V\mathrm{_{cmax25}} \end{equation*}.

We used this simple model to predict *N*_area_ first from observed—and then from predicted—*V*_cmax25_ and *M*_a_. In this way, we could first test whether *N*_area_ is indeed predictable from *V*_cmax25_ and *M*_a_ in our data set and then test whether *N*_area_ is predictable from the climate data alone. In order to examine the impact of nitrogen fixation on this relationship, we also included ‘N-fixer’ as a factor in this linear model. Partial residuals from the regression model for *N*_area_ were plotted using the *visreg* package (Breheny and Burchett 2017).

#### Estimating the contribution of individual predictor variables

The contribution of each predictor variable to trait variation was calculated in three steps. At Step 1, we created a baseline by averaging the values of each predictor variable across the 18 sites to create a data set for an ‘average’ site. We used this average site data to calculate baseline trait values. At Step 2, we changed one predictor variable at a time to the actual value at that site, keeping all the other variables constant at the average site value. We then calculated trait values using these new inputs. At Step 3, the contribution of each predictor variable was calculated as the difference between the traits simulated at Step 2 and the baseline value of the traits from Step 1. This procedure allowed us to separate out the individual influences of changes in air pressure with elevation, *T*_dJ_ and *D*_0_ on χ, the influence of changes in air pressure with elevation, *T*_dJ_ and *R*_0_ on *V*_cmax25_, as well as the impact of χ itself on *V*_cmax25_. It also allowed us to separate the effects of *T*_g_ and *R*_LAI_ on *M*_a_ and the effects of LL (indexed by gsl) and moisture (indexed by the ratio of annual actual evapotranspiration to annual potential evapotranspiration) on *M*_a_.

#### Uncertainty of the model predictions

The uncertainty of trait prediction can come from two sources: parameter values and input data. To evaluate the parameter uncertainty, we calculated the uncertainty of each parameter separately and combined them using the standard error propagation formula:

(20) \begin{equation*} {u}^2(y)={\sum}_{\mathrm{i}}{\left(\frac{\partial m}{{\partial n}_i}\right)}^2{u}^2\left({n}_{\mathrm{i}}\right), \end{equation*}

where *u*(*y*) is the standard uncertainty of the trait, *∂m/∂n*_i_ is the sensitivity to variable *n*_i_ (obtained by differentiating the individual equations) and *u*(*n*_i_) is the standard uncertainty of *n*_i_. The uncertainty of predicted *M*_a_ and *N*_area_ values arises from the uncertainties in the coefficients fitted by regression and additional observed *M*_a_ and *V*_cmax25_ for *N*_area_. The uncertainty of χ and *V*_cmax25_ arises from the values of the various ecophysiological quantities in the prediction equations and additional observed χ for *V*_cmax25_, which show some degree of variation among species.

### Model evaluation

We evaluated model performance by comparing the observed mean trait value at each site with predictions of each trait, using *r* and root mean square error (RMSE) between the observed and predicted values across the sites. We compared the *R*^2^ explained by the optimality models and statistical models. To test whether the optimality-based models can capture the climate variability, we also fitted multiple linear regressions of the site-mean trait values against the driving climate data which serve as a statistical benchmark. All statistics were performed in R3.1.3.

## Results

### Trait variation related to climate

**Figure 2.**
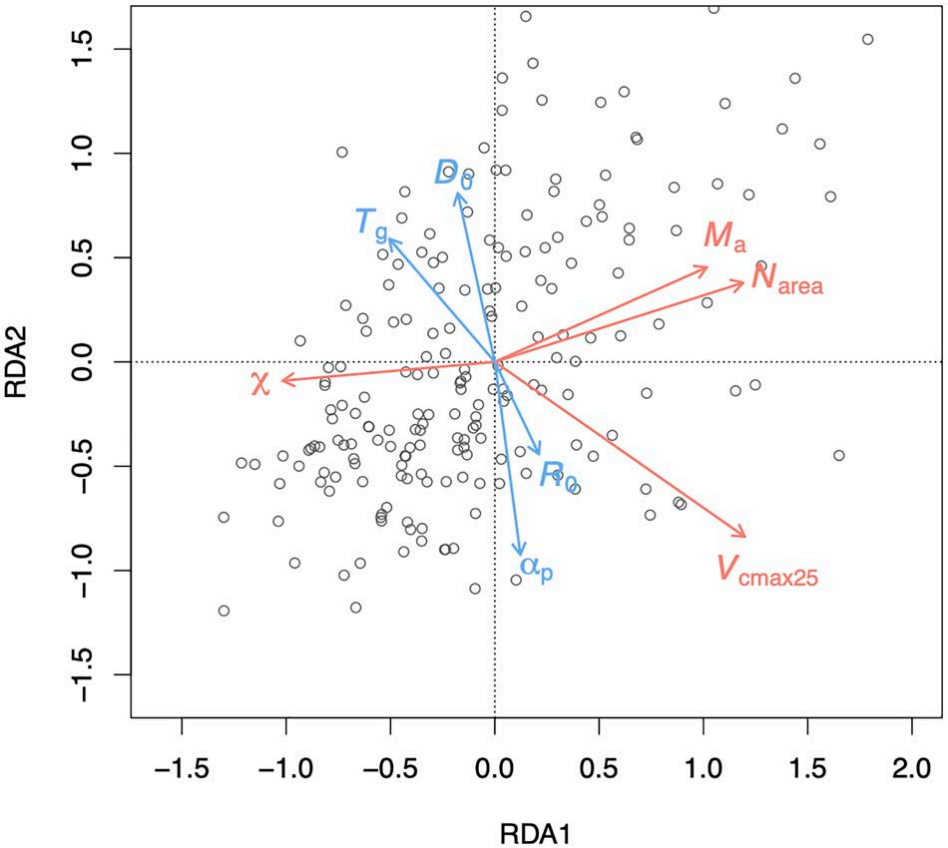
Climate-related trait dimensions from redundancy analysis (RDA). The climate variables (shown by blue arrows) are mean temperature during the growing season, defined as days above a baseline of 0 °C (*T*_g_), mean vapor pressure deficit (*D*_0_), mean photosynthetically active radiation (*R*_0_) and a moisture index (α_p_) defined as the ratio of annual actual evapotranspiration to annual potential evapotranspiration. The traits (shown by red arrows) are leaf mass per area (*M*_a_), leaf nitrogen content per area (*N*_area_), the maximum capacity of carboxylation standardized to 25 °C (*V*_cmax25_) and the ratio of leaf-internal to ambient CO_2_ partial pressures (χ). The gray circles are species average values from each site.

The four climate variables together accounted for 22.2% of the trait variation as shown in the RDA. The first axis explained 16.9% of the variability in the observations. On this axis, variability was negatively related with temperature and positively related with *R*_0_ (Figure 2). The second axis reflected gradients in moisture (α_p_ and vapor pressure deficit). Variability in χ was shown to be controlled by moisture, although with a small influence from temperature. The *V*_cmax25_ varied positively with radiation, and negatively with temperature and moisture, in the opposite direction from χ. Temperature had a small positive influence on *M*_a_ but moisture had a negative impact, reflecting the fact that leaves were thicker in hotter and drier environments. The *N*_area_ was mainly controlled by radiation and moisture and covaried with *M*_a_ and *V*_cmax25_.

### Observed and predicted trait variation with elevation

**Figure 3.**
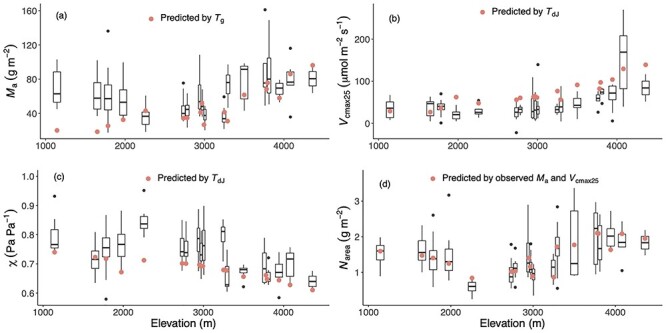
The observed and predicted values of traits along the altitudinal gradient. The traits are leaf mass per area (*M*_a_), leaf nitrogen content per unit area (*N*_area_), the maximum capacity of carboxylation standardized to 25 °C (*V*_cmax25_) and the ratio of leaf-internal to ambient CO_2_ partial pressure (χ). Only the observed trait values of deciduous plants are shown in black with box plots. The best versions of each predicted trait are shown as red dots: predicted *M*_a_ using mean temperature during the growing season, defined as days above a baseline of 0 °C (*T*_g_), predicted *V*_cmax25_ and χ driven by daily temperature in July (*T*_dJ_) and predicted *N*_area_ using observed *M*_a_ and *V*_cmax25_.

**Figure 4.**
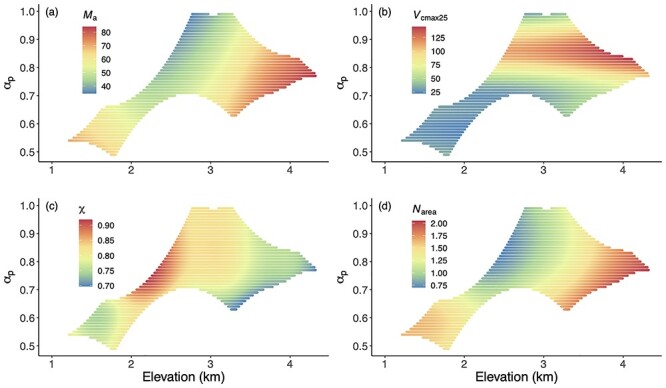
The distribution of predicted trait values in a climate space defined by elevation and a moisture index (α_p_) using GAMs. The traits are (a) leaf mass per area (*M*_a_), (b) the maximum capacity of carboxylation standardized to 25 °C (*V*_cmax25_), (c) the ratio of leaf-internal to ambient CO_2_ partial pressure (χ) and (d) leaf nitrogen content per unit area (*N*_area_). Trait values are indicated by the color scale.

All observed traits showed non-linear relationships with elevation (Figure 3). Trait distributions in climate space also showed non-linear relationships. (Figure 4). These non-linear relationships arose because although temperature (as measured by either *T*_g_ or *T*_dJ_) decreased monotonically with elevation, the moisture-related variables in the Gongga Mountain region had non-linear relationships with elevation (Figure 1c): the lowest and uppermost sites had lower mean annual precipitation (MAP) and α_p_ than the sites at intermediate elevations (see Table S1 available as Supplementary data at *Tree Physiology* online). The combination of these different trends in individual climate variables led to a complex pattern of trait variability. The *M*_a_ and *N*_area_ were large under dry conditions and high elevation. The *V*_cmax25_ increased along elevation and moisture gradients. The χ was lower under dry conditions and low elevation. Nevertheless, *M*_a_, *V*_cmax25_ and *N*_area_ tended to increase overall with elevation, while χ showed an overall decrease with elevation. There was no trend in the CV of any of the traits with elevation (see Figure S3 available as Supplementary data at *Tree Physiology* online). Within-site CV values were larger than across-site CV values at nearly half of the sites for *M*_a_, χ and *N*_area_, while most of the within-site CV values were smaller than across-site CV values for *V*_cmax25_. However, within-site variability differed between the traits. The *V*_cmax25_ was the most and χ was the least variable trait.

**Figure 5.**
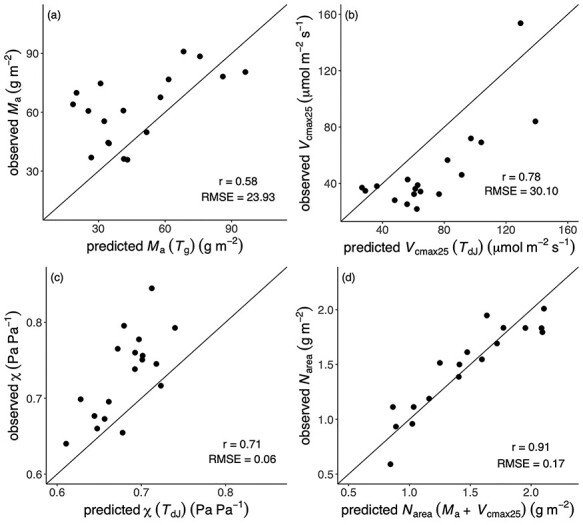
Site-mean values of traits. The traits are (a) leaf mass per area (*M*_a_), (b) the maximum capacity of carboxylation standardized to 25 °C (*V*_cmax25_), (c) the ratio of leaf-internal to ambient CO_2_ partial pressure (χ) and (d) leaf nitrogen content per unit area (*N*_area_). Observations are site-mean values and predictions are the best versions of different driven data at each site: predicted *M*_a_ using mean temperature during the growing season (*T*_g_), predicted *V*_cmax25_ and χ driven by daily temperature in July (*T*_dJ_) and predicted *N*_area_ using observed *M*_a_ and *V*_cmax25_. The solid line is the 1:1 line.

The models captured the overall patterns of variability of the four traits between sites, with most of predicted values falling within the range of the observed values. The observed and predicted site-mean values followed the 1:1 line (Figure 5), and the average of the *r* values for the four traits was 0.75. Mean RMSE values showed that differences between observations and predictions accounted for close to 30% of the mean trait values. The *R*^2^ values produced by the optimality models were generally higher, except for *M*_a_, due to its underestimation at low elevation (Table 2). The models also captured χ and *V*_cmax25_ variations for evergreen species, with *r* values of 0.68 and 0.67, respectively (see Figure S4 available as Supplementary data at *Tree Physiology* online). However, predicted *M*_a_ using *T*_dJ_, χ using *T*_g_ and predicted *N*_area_ using *N*_structure_ and *N*_rubisco_ were underestimated, and *V*_cmax25_ using *T*_g_ was overestimated (see Figure S5 available as Supplementary data at *Tree Physiology* online). Using *T*_dJ_ instead of *T*_g_ improved the predictions of *V*_cmax25_ and χ but degraded the prediction for *M*_a_ (Figure 5, see Figure S6 available as Supplementary data at *Tree Physiology* online). The predicted χ values using *T*_dJ_ were better than those using *T*_g_, and the best-fit model could predict the values across the sites with *r* = 0.71 and RMSE = 0.06 despite the bias, with median values of χ underpredicted at most sites (Figure 3). The uncertainties of predicted *V*_cmax25_ and *N*_area_ were much narrower than the observed ranges. All parameters in the *N*_area_ models contributed almost equally to the uncertainty, while the parameter *c* was the major source of uncertainty for *V*_cmax25_. The large uncertainty of *M*_a_ and χ mainly resulted from the intercept and the parameter β, respectively (see Figure S7 available as Supplementary data at *Tree Physiology* online).

**Table 2:** The comparison between *R*^2^ of statistical models (multiple linear regressions of the site-mean trait values against the driving climate data) and optimality models. For *M*_a_, *V*_cmax25_ and χ, ‘*T*_g_’ in parentheses represents the predicted traits using mean temperature during growing season, ‘*T*_dJ_’ represents the predicted traits using daytime temperature in July. For *N*_area_, ‘*M*_a_ + *V*_cmax25_’ represents the predicted *N*_area_ using observed *M*_a_ and *V*_cmax25_ in Eq. (19).

Traits	Statistical model	Optimality model
*M*_a_ (*T*_g_)	0.55	0.33
*V*_cmax25_ (*T*_dJ_)	0.45	0.60
χ (*T*_dJ_)	0.49	0.51
*N*_area_ (*M*_a_ + *V*_cmax25_)	0.65	0.84

**Figure 6.**
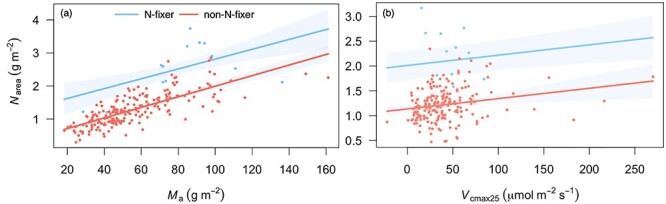
Partial residual plots showing leaf nitrogen content per unit area (*N*_area_) as a function of leaf mass per area (*M*_a_) and the maximum capacity of carboxylation standardized to 25 °C (*V*_cmax25_) with nitrogen-fixer as an interaction term. (a) The *N*_area_ as a function of *M*_a_ and (b) *N*_area_ as a function of *V*_cmax25_. Blue, nitrogen-fixing plants (N-fixer); red, non-nitrogen-fixing plants (non-N-fixer).

The *N*_area_ was shown to be strongly correlated with both *M*_a_ and *V*_cmax25_ (*P* < 0.001) (Figure 6, see Figure S8 available as Supplementary data at *Tree Physiology* online). However, there was a significant effect of including nitrogen fixation (‘N-fixer’) as a factor. At any given *M*_a_ or *V*_cmax25_, *N*_area_ was slightly higher in the nitrogen-fixing species. The prediction of *N*_area_ directly from *M*_a_ and *V*_cmax25_ with our simple method (Eq. 19) was marginally closer to the data than the prediction from *M*_a_ and *V*_cmax25_ via *N*_structure_ and *N*_rubisco_ (see Figure S5 available as Supplementary data at *Tree Physiology* online). The predicted site-mean *N*_area_ with our new method but from predicted *M*_a_ and *V*_cmax25_ was also not significantly different from the observed *N*_area_ (*P* = 0.08). These ‘fully predicted’ *N*_area_ values were within the range of observations at most sites but were underestimated at low elevation due to the underestimation of predicted *M*_a_ (see Figure S5 available as Supplementary data at *Tree Physiology* online).

### Contribution of climate and elevation to trait variations

**Figure 7.**
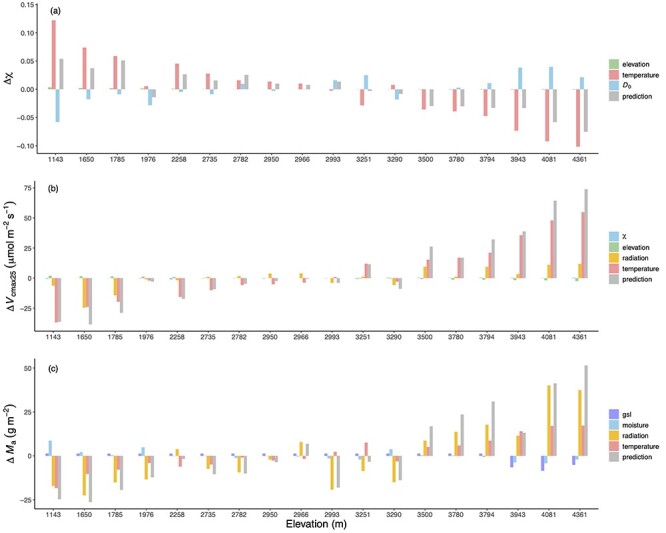
The modeled contributions of individual climate variables for each trait at each site. The traits are (a) the ratio of leaf-internal to ambient CO_2_ partial pressures (χ), (b) the maximum capacity of carboxylation standardized to 25 °C (*V*_cmax25_) and (c) leaf mass per area (*M*_a_). The gray bars show the changes in predicted trait values compared with the reference level driven by site-mean environment. The green bars show the elevation effect on χ and *V*_cmax25_ due to the changes in air pressure. The red bars show the effects of average daytime temperature in July on χ and *V*_cmax25_ and the effect of growing season mean temperature on *M*_a_, respectively. The blue bars show the effect of vapor pressure deficit (*D*_0_) on χ and then the effect of χ on *V*_cmax25_. The yellow bars show the effect of growing season mean radiation on *V*_cmax25_ and the effect of leaf-area-index-weighted growing season mean radiation on *M*_a_, respectively. The effects of LL (indexed by gsl) and moisture (indexed by the ratio of annual actual evapotranspiration to annual potential evapotranspiration) on *M*_a_ are shown in purple and blue.

Vapor pressure deficit and temperature were shown to be the most important factors influencing the variation in χ between sites at different elevations in the Gongga Mountain region, but with opposing effects. Elevation made little contribution to the variation of χ. The *V*_cmax25_ was influenced most by temperature and radiation, but elevation also had a small impact on *V*_cmax25_. The effects of all the predictors were important for *M*_a_ (Figure 7).

## Discussion

Optimality models have shown skill in predicting the trait variations along the elevation gradient in the Gongga Mountain region, without site- or region-specific calibration of parameters. The *r* of optimality models was generally higher than statistical models (Table 2). The *r* of the optimal *M*_a_ model was 0.73 when four predictions at low elevation were excluded. This finding suggests that the optimality models considering the underlying mechanisms are better than the statistical models and supports the general validity of these models. The new model for *M*_a_—calibrated using an independent set of measurements—correctly predicted patterns in the community-mean *M*_a_ of deciduous plants at the Gongga Mountain sites. When the coefficients of *I*_abs_, *f*, *T*_g_ and α_p_ were calibrated with the sampled data, the values obtained were 0.99, 0.52, −0.03 and − 0.75, which were not significantly different from the values obtained using the China Plant Trait database but were closer to the values for *I*_abs_ and *T*_g_ deduced from the theory. We did not apply the new model to evergreen species because we had no information about their LL. Leaf longevity is strongly related to *M*_a_ (Kikuzawa 1991, Reich et al. 1997, Wright and Westoby 2002, Wright et al. 2004). According to the leaf economics spectrum, the LL and *M*_a_ of deciduous plants are smaller than those of evergreen plants (Wright et al. 2004). However, LL cannot be reliably estimated in the field without monitoring over a long period (Cornelissen et al. 2003). If such data were available, it would be possible to extend the *M*_a_ model to evergreen species.

We have developed a simplified approach to predict *N*_area_. This approach produced results close to those obtained using the two-step approach put forward by Dong et al. (2017). The agreement between predictions using the two methods suggests that the hypothesis underpinning both, namely that *N*_area_ can be predicted as the sum of a photosynthetic component related to *V*_cmax25_ and a non-photosynthetic component proportional to *M*_a_ (Evans 1989, Lambers and Poorter 1992, Onoda et al. 2004), is reasonable. However, our simpler approach does not require explicit specification of the relative allocation to the metabolic and structural components and, by removing the intermediate steps, reduces the uncertainties and improves the predictions. We have shown that *M*_a_ and *V*_cmax25_ are predictable from climate and that fully predicted *N*_area_ values lie within the range of observations at most sites (see Figure S5 available as Supplementary data at *Tree Physiology* online). This interpretation differs from some previous studies in which leaf nitrogen availability, implicitly assumed to be related to soil nitrogen availability, is used to predict *V*_cmax25_ (Luo et al. 2004). There is evidence that soil nutrients, particularly phosphorus rather than nitrogen, can influence *N*_area_ and *V*_cmax25_ (He et al. 2014, Gvozdevaite et al. 2018). However, there is growing evidence (i) that LMA exerts a major control on *N*_area_ (Dong et al. 2017) and (ii) that climate variables are the dominant drivers of *V*_cmax_. Smith et al. (2019) found that climate variables accounted for about two-thirds of global variation in *V*_cmax_; soil fertility indices accounted for about one-third. Liang et al. (2020), in a meta-analysis of soil nitrogen enhancement experiments, showed a 2–4 times greater effect on the leaf area and biomass (i.e., whole-plant carbon allocation responses) than on leaf-level *N*_area_ and *V*_cmax_. In other words, consistent with optimality theory, plants react to nutrient deficiency more by reducing leaf area, and increasing below-ground carbon investment, than by developing suboptimal leaves. Thus, a key implication of our results is that leaf nitrogen content can be predicted from climate alone. No global analysis of *N*_area_ is yet available, but the consistency of results for Australia (Dong et al. 2017), Peru (Peng et al. 2020) and this study strongly supports the idea. Moreover, further work should focus on improving *M*_a_ prediction since fully predicted *N*_area_ is underestimated at low elevation due to the underestimation of *M*_a_. We have also shown that *R*_0_ is positively related to *N*_area_—consistent with widespread observations that leaf nitrogen is higher at the top of the canopy (Hirose and Werger 1987, Chen et al. 1993) and the optimality hypothesis that nitrogen is unequally allocated within the canopy so as to maximize photosynthesis at each canopy level (Werger and Hirose 1991, Peltoniemi et al. 2012).

Our analyses provide insights into the timescales on which leaf trait acclimation and adaptation operate. Since optimality models implicitly consider acclimation and adaptation in physiological processes, the use of climate inputs at the appropriate timescale—which resulted in better predictions—might provide insight on the corresponding adaptation/acclimation timescale of a trait. We showed that *T*_g_ was a better predictor than *T*_dJ_ for *M*_a_, suggesting that *M*_a_ adapts to the whole growing season environment. The adaptation of *M*_a_ to long-term temperature is consistent with the fact that deciduous leaves are built at the beginning of the growing season with one-time carbon investment from the previous year and maximize average carbon gain per day, and in turn, net carbon gain during the whole growing season (Kikuzawa 1991). However, although predictions of *V*_cmax25_ have commonly been made using long-term temperature inputs such as *T*_g_ (Wang et al. 2017*a*, Smith et al. 2019), our results show this can lead to a mis-estimation of *V*_cmax25_. Using *T*_dJ_ (i.e., daytime during the month the plants were sampled) gives a better prediction, suggesting that *V*_cmax25_ adapts to environmental conditions during the previous few weeks. Several studies have shown that photosynthetic traits can acclimate quickly to temperature changes (Smith and Dukes 2017, Smith et al. 2017) by regulating intrinsic biochemical characteristics, such as Rubisco content or catalytic turnover rate (Cavanagh and Kubien 2014). Our model data comparison also suggests that χ acclimates to *T*_dJ_ rather than *T*_g_. The least-cost hypothesis underlying the model of χ considers the total cost of maintaining plant carboxylation and transpiration. Both metabolic processes function mainly in the daytime and can be adjusted rapidly. Therefore, the regulation of χ is also expected to acclimate to daytime temperature at a weekly to monthly scale, consistent with our finding that χ is better predicted using *T*_dJ_ than *T*_g_. The χ is highly plastic compared with *M*_a_ (Dong et al. 2017), and seasonal variations in χ for deciduous species have been observed in many studies (Chen and Chen 2007, Ma et al. 2010, McKown et al. 2013); however, the correlation of leaf phenology with seasonal changes in the growth environment of deciduous leaves indicates a need to disentangle their effects in the future. Given that different processes have different timescales for acclimation/adaptation, model inputs should be selected to reflect this.

We have focused on predicting community-mean trait values. Although between-site variation is larger than within-site variation for all traits, nevertheless, there is considerable variability at each site. This variability presumably reflects the within-canopy heterogeneity in bioclimate and in particular in radiation. There are large differences in the photosynthetic traits between sunlit and shaded leaves, and it has also been shown that sunflecks contribute greatly to the photosynthesis of shaded leaves. Our model for *M*_a_ is sensitive to radiation inputs. By using *R*_LAI_ to estimate the average light level absorbed by the leaves within the canopy to drive the *M*_a_ model, we were able to obtain relatively good predictions of the community-mean values except at the lowest sites, which may be attributable to disturbance, since many people live at lower elevations in this region. This approach would be insufficient to model within-canopy variability. However, site-based radiation measurements could be used in order to test whether this optimality-based model could predict within-site variation, given appropriate inputs. The within-canopy heterogeneity of other bioclimatic factors may also be important in the choice of appropriate model inputs (Blonder et al. 2018) and for testing the applicability of optimality-based models to explain the within-site variability.

The comparison between the observed and simulated traits allows us to identify mechanisms that are missing from the current optimality framework. For example, our analysis emphasizes the importance of soil moisture constraints. The RDA showed that *V*_cmax25_ was positively associated with soil moisture, indexed by α_p_. We found significant relationships between α_p_ and the residuals of predicted χ and *V*_cmax25_. Some hydraulic traits, including the ratio of leaf-to-sapwood area and specific sapwood hydraulic conductance, also showed significant correlations with photosynthetic traits (see Figure S9 available as Supplementary data at *Tree Physiology* online), suggesting coordination between photosynthesis and water transport. Many studies have shown a strong coordination between hydraulic and photosynthetic traits across species (Brodribb 2009, Scoffoni et al. 2016, Zhu et al. 2018), especially when the hydraulic structure plays a crucial role in limiting the photosynthesis process under water stress (Tyree and Sperry 1989). Lin et al. (2015) analyzed a large global data set and found a positive relationship between wood density and carbon cost per unit water use. We have detected a significant positive effect of wood density on *V*_cmax25_. Further empirical analysis on the coordination between photosynthetic and hydraulic traits over a larger environmental gradient is required. The coordination of photosynthesis and hydraulic traits has already been considered in models to predict stomatal response (Sperry et al. 2017) and vegetation response to drought (Eller et al. 2018), and has been shown to produce improved predictions under water-limited conditions. Our results underline the need to consider aspects of water limitation, in addition to the stomatal response to vapor pressure deficit, in order to predict key plant traits.

Empirical analyses have shown that LL is positively related to potential evapotranspiration and vapor pressure deficit (Wright et al. 2004). In our model, to predict *M*_a_, the effect of α_p_ was based on an empirical analysis of an independent global trait data set because there is currently no theory to explain the impact of moisture on optimal LL. Using local data to calibrate the parameters for the theoretical model of *M*_a_ showed that the estimated effect of α_p_ is stronger than that indicated by the China Plant Trait database. The RMSE of predictions using the two different sets of calibrated parameters showed larger differences in the lowest values, where the soil moisture constraint is more severe. Given that the effects of other climate variables on *M*_a_ are well captured by the model, it would be worthwhile to try to identify and incorporate the mechanism of moisture impact on optimal LL.

The large functional diversity within sites may result from species attributes, biotic factors or microenvironment (Violle et al. 2014, Pappas et al. 2016). The model uncertainty analysis may provide a new way to estimate the functional diversity. Uncertainty analysis showed that the parameters β and *c*, representing unit costs for the maintenance of carboxylation, electron transport and transpiration, are the main contributors to uncertainty in χ and *V*_cmax25_, respectively (see Figure S7 available as Supplementary data at *Tree Physiology* online). Empirical analysis has shown substantial interspecific variation in β, but the current model of χ uses a single value of β for all species (Wang et al. 2017*b*). Using a single value estimated from the published values of photosynthetic capacity (Kattge and Knorr 2007, Wang et al. 2017*b*) for the parameter *c* in the model of *V*_cmax25_, similarly, cannot fully represent its variation among species. Predictions using average values of β and *c* estimated from published data could cause mismatches with observed values, such as the predicted χ being lower than median observed value at many sites (Figure 3). At the same time, parameter uncertainty due to species variation also represents functional diversity in the community, which could in principle be considered in ecosystem models by specifying a realistic range of values for each parameter. Meanwhile, modeling functional diversity still needs further work both in theory and application.

### Implications for terrestrial ecosystem models

Optimality theory relies on the concept that natural selection requires plants to acclimate or adapt to prevailing environmental conditions. The development of optimality-based models therefore focuses on identifying the trade-offs between competing requirements. We have shown that optimality-based models for four key traits related to photosynthesis, *M*_a_, *N*_area_, *V*_cmax_ and χ, predict community-level variability with elevation and climate in the Gongga region, with no need for site- or regional-scale calibration. This finding adds to the growing number of studies showing that patterns of variation in these traits along climate gradients are predictable (Meng et al. 2015, Wang et al. 2017*a*).

Optimality-based models could be beneficially incorporated into vegetation- or land-surface models since they provide a natural way of accounting for trait variability within PFTs, or across vegetation types, as a function of environmental gradients. The prediction of continuous trait variation with environment would obviate the need to specify parameter values separately for different PFTs (Kucharik et al. 2000, Sitch et al. 2003, Kim et al. 2018) or to account for within-PFT variability probabilistically (see e.g., Kelley et al. 2014). Moving from PFT-based parameters to optimality-based formulations would have the desirable effect of reducing the number of parameters that have to be specified. Moreover, models should improve in realism if the parameter values are allowed to adjust to changing environmental conditions.

However, some issues need to be addressed before implementing optimality-based trait models into vegetation models. First, the timescales of acclimation and adaptation differ between traits. Thus, it is important to ensure that the variability of a given trait is predicted using the appropriate climate information, for example, daytime temperature over a week or month (rather than a climatological growing season average) in the case of *V*_cmax25_. Second, although soil moisture can limit photosynthesis, we lack theoretical understanding of the coordination between plant photosynthesis and hydraulics required to account for this constraint within the current optimality-based modeling framework. Third, the current framework does not account for within-site trait variability and thus does not account for functional diversity within communities. Nevertheless, our study suggests a promising way forward to improve both the robustness (with fewer parameters) and realism (considering the acclimation and adaptation of traits) of terrestrial ecosystem models through the prediction of continuous trait variation along environmental gradients.

## Supplementary Material

Supplementary_tpab003

## Data Availability

The trait and climate data are available from Zenodo (Xu et al. 2020). The codes to produce the predicted trait values along with a readme file can be found on GitHub (https://github.com/Huiying-Xu/PTG).
