# Supplementary material for: Predictability of leaf traits with climate and elevation: a case study in Gongga Mountain, China
Source: Tree Physiol. 2021 Jan 13;41(8):1336–52. doi: 10.1093/treephys/tpab003 (PMC8454210; doi:10.1093/treephys/tpab003)

**Predictability of leaf traits with climate and elevation: a case study in Gongga Mountain, China: Supplementary Information**

Huiying Xu^1,2^, Han Wang^1,2^, I. Colin Prentice^1,3,4^, Sandy P. Harrison^1,5^, Genxu Wang^6^, Xiangyang Sun^6^

^1^Ministry of Education Key Laboratory for Earth System Modeling, Department of Earth System Science, Tsinghua University, Beijing 100084, China;

^2^Joint Center for Global Change Studies (JCGCS), Beijing 100875, China;

^3^Department of Life Sciences, Imperial College London, Silwood Park Campus, Buckhurst Road, Ascot, SL5 7PY, UK;

^4^Department of Biological Sciences, Macquarie University, North Ryde, NSW 2109, Australia;

^5^School of Archaeology, Geography and Environmental Sciences (SAGES), University of Reading, Reading, RG6 6AH, UK;

^6^Institute of Mountain Hazards and Environment, Chinese Academy of Sciences Institute of Geographic Sciences and Natural Resources Research, Chinese Academy of Sciences

**Table S1. Characteristics of the study sites.** Climate data: mean temperature during the growing season (*T*_g_), the ratio of growing season length to the number of days in the year (*f*), mean photosynthetically active radiation (*R*_0_), mean vapor pressure deficit (*D*_0_), mean annual precipitation (MAP), ambient partial pressure of O_2_ (*P*_0_), moisture index (α_p_) and leaf area index (LAI). The sample size gives the number of species sampled at each site.

| Geographic information | | | Climate information | | | | | | | | Vegetation type | Sample size |
| --- | --- | --- | --- | --- | --- | --- | --- | --- | --- | --- | --- | --- |
| Elevation (m) | Longitude  (°E) | Latitude  (°N) | *T*_g_  (˚C) | *f*  (day day^–1^) | *R*_0_  (μmol m^–2^ s^–1^) | *D*_0_ (kPa) | MAP  (mm) | α_p_  (mm mm^–1^) | *P*_0_  (Pa) | LAI  (m^2^ m^-2^) |  |  |
| 1143 | 102.16 | 29.43 | 20.33 | 1 | 293 | 1.18 | 1046 | 0.53 | 18589 | 3.7 | Deciduous broad-leaved forest | 27 |
| 1650 | 102.13 | 29.65 | 15.89 | 1 | 202 | 0.81 | 994 | 0.66 | 17508 | 2.6 | Deciduous broad-leaved forest | 25 |
| 1785 | 102.10 | 29.65 | 14.77 | 1 | 253 | 0.74 | 1292 | 0.70 | 17229 | 2.6 | Deciduous broad-leaved forest | 21 |
| 1976 | 101.02 | 29.38 | 13.14 | 1 | 319 | 0.89 | 821 | 0.59 | 16840 | 3.6 | Deciduous broad-leaved forest | 29 |
| 2258 | 102.05 | 29.59 | 14.01 | 1 | 316 | 0.72 | 657 | 0.72 | 16279 | 1.7 | Deciduous broad-leaved forest | 26 |
| 2735 | 101.91 | 29.42 | 13.50 | 1 | 330 | 0.74 | 1396 | 0.71 | 15365 | 3.0 | Deciduous broad-leaved forest | 28 |
| 2782 | 102.03 | 29.59 | 11.92 | 1 | 333 | 0.62 | 1500 | 0.74 | 15277 | 3.3 | Deciduous broad-leaved forest | 36 |
| 2950 | 101.92 | 29.37 | 12.57 | 1 | 343 | 0.70 | 906 | 0.72 | 14966 | 2.6 | Deciduous broad-leaved forest | 27 |
| 2966 | 101.88 | 29.45 | 12.21 | 1 | 344 | 0.69 | 1113 | 0.72 | 14937 | 1.8 | Deciduous broad-leaved forest | 29 |
| 2993 | 102.00 | 29.57 | 10.73 | 1 | 305 | 0.58 | 1568 | 0.75 | 14888 | 4.3 | Deciduous broad-leaved forest | 21 |
| 3251 | 101.99 | 29.57 | 8.97 | 1 | 330 | 0.53 | 1660 | 0.77 | 14423 | 3.2 | Evergreen needle-leaved forest | 10 |
| 3290 | 101.39 | 29.55 | 12.76 | 1 | 295 | 0.81 | 1145 | 0.62 | 14354 | 3.4 | Deciduous broad-leaved forest | 20 |
| 3500 | 101.53 | 29.47 | 9.78 | 1 | 371 | 0.68 | 935 | 0.70 | 13986 | 2.1 | Deciduous broad-leaved forest | 18 |
| 3780 | 101.60 | 29.45 | 9.51 | 1 | 330 | 0.66 | 1108 | 0.71 | 13507 | 1.2 | Evergreen needle-leaved forest | 17 |
| 3794 | 101.66 | 29.52 | 8.61 | 1 | 371 | 0.61 | 1047 | 0.73 | 13483 | 1.5 | Deciduous shrub | 15 |
| 3943 | 102.00 | 29.90 | 7.03 | 0.79 | 341 | 0.46 | 1576 | 0.82 | 13234 | 1.5 | Deciduous shrub | 9 |
| 4081 | 102.01 | 29.91 | 6.20 | 0.74 | 379 | 0.46 | 887 | 0.84 | 13007 | 0.5 | Deciduous shrub | 17 |
| 4361 | 101.71 | 29.52 | 6.14 | 0.82 | 383 | 0.55 | 1333 | 0.77 | 12555 | 0.7 | Evergreen shrub | 13 |

**Table S2. Species sampled at each site.** The list provides information on the plant functional type and the species on which measurements were made.

| Elevation | Plant functional type | Species sampled |
| --- | --- | --- |
| 1143 | deciduous broadleaf tree | *Euptelea pleiosperma, Betula utilis, Juglans cathayensis* |
|  | deciduous broadleaf small tree | *Pyracantha fortuneana, Viburnum foetidum var. ceanothoides, Litsea cubeba, Quercus gilliana, Pistacia weinmannifolia* |
|  | evergreen broadleaf tree | *Cunninghamia lanceolata, Cyclobalanopsis glaucoides* |
|  | evergreen broadleaf small tree | *Rhamnus dumetorum, Trachycarpus fortunei, Ilex corallina, Hydrangea xanthoneura* |
|  | deciduous broadleaf shrub | *Debregeasia orientalis, Rubus macilentus, Grewia biloba* |
|  | evergreen broadleaf shrub | *Myrsine semiserrata* |
|  | liana | *Clematis grandidentata* |
|  | forb | *Boehmeria clidemioides var. diffusa, Elatostema cuspidatum, Arisaema erubescens, Begonia henryi, Boenninghausenia albiflora, Lophatherum gracile* |
|  | pteridophyte | *Woodwardia unigemmata* |
| 1650 | deciduous broadleaf tree | *Alnus cremastogyne, Quercus serrata var. brevipetiolata, Rhus chinensis, Toxicodendron sylvestre* |
|  | deciduous broadleaf small tree | *Rhamnus tangutica, Morus australis, Alangium chinense* |
|  | evergreen broadleaf tree | *Cyclobalanopsis glaucoides, Ligustrum lucidum* |
|  | evergreen broadleaf small tree | *Lyonia ovalifolia var. lanceolata, Rhododendron augustinii* |
|  | deciduous broadleaf shrub | *Debregeasia orientalis, Neillia affinis, Lespedeza formosa, Rosa glomerata* |
|  | evergreen broadleaf shrub | *Pyracantha fortuneana, Maclura tricuspidata* |
|  | liana | *Clematis smilacifolia var. peltata* |
|  | forb | *Agrimonia pilosa, Arisaema erubescens, Zingiber striolatum, Artemisia argyi, Anaphalis bicolor, Lophatherum gracile* |
|  | pteridophyte | *Pseudocyclosorus esquirolii* |
| 1785 | deciduous broadleaf tree | *Alnus ferdinandi-coburgii, Betula utilis, Tetracentron sinense, Salix wallichiana, Rhus chinensis* |
|  | deciduous broadleaf small tree | *Litsea cubeba* |
|  | evergreen broadleaf small tree | *Viburnum oliganthum* |
|  | deciduous broadleaf shrub | *Coriaria nepalensis, Indigofera szechuensis, Elaeagnus umbellata, Rubus mesogaeus, Debregeasia orientalis, Cotoneaster dielsianus, Aster albescens, Pyracantha fortuneana, Lonicera ligustrina, Salix variegata, Hydrangea strigosa, Rubus setchuenensis, Rubus lambertianus, Ficus tikoua* |
|  | evergreen broadleaf shrub | *Viburnum atrocyaneum, Viburnum rhytidophyllum* |
|  | liana | *Stauntonia chinensis, Berchemia floribunda, Galium dahuricum var. lasiocarpum, Paederia foetida, Clematis pogonandra, Fallopia multiflora* |
|  | forb | *Arisaema erubescens, Begonia grandis, Pilea pumila, Artemisia tangutica, Incarvillea arguta* |
| 1976 | deciduous broadleaf tree | *Alnus cremastogyne, Juglans cathayensis, Euptelea pleiosperma, Machilus viridis, Toxicodendron vernicifluum* |
|  | deciduous broadleaf small tree | *Elaeagnus bockii, Salix wallichiana, Styrax roseus, Sorbaria arborea, Litsea cubeba, Viburnum betulifolium* |
|  | deciduous broadleaf shrub | *Rubus lambertianus, Rhamnus dumetorum, Hydrangea anomala, Rubus setchuenensis, Neillia affinis, Cotoneaster dielsianus* |
|  | liana | *Viburnum foetidum var. rectangulatum, Clematoclethra scandens subsp. actinidioides* |
|  | forb | *Iris confusa, Zingiber striolatum, Agrimonia pilosa, Artemisia argyi, Boehmeria clidemioides var. diffusa,*  *Oplismenus undulatifolius, Carex henryi, Bambusa multiplex, Arisaema erubescens* |
|  | pteridophyte | *Dryopteris neorosthornii* |
| 2258 | deciduous broadleaf tree | *Malus prattii, Euptelea pleiosperma, Sorbus meliosmifolia, Tetracentron sinense* |
|  | deciduous broadleaf small tree | *Corylus ferox, Cornus controversa, Ilex fragilis f. kingii* |
|  | evergreen broadleaf tree | *Lithocarpus cleistocarpus, Machilus viridis* |
|  | evergreen broadleaf small tree | *Rhododendron polylepis, Ilex pernyi, Ilex yunnanensis* |
|  | deciduous broadleaf shrub | *Polygala fallax, Rubus pentagonus, Ribes longiracemosum, Viburnum kansuense* |
|  | evergreen broadleaf shrub | *Viburnum oliganthum* |
|  | liana | *Clematoclethra scandens subsp. actinidioides* |
|  | forb | *Adenocaulon himalaicum, Pternopetalum davidii, Oxalis griffithii, Rubus fockeanus, Calanthe tricarinata, Carex henryi, Fargesia ferax* |
|  | pteridophyte | *Polystichum braunii* |
| 2735 | deciduous broadleaf tree | *Sorbus pallescens, Tilia chinensis var. intonsa, Padus buergeriana, Acer laxiflorum, Betula utilis* |
|  | deciduous broadleaf small tree | *Lonicera lanceolata, Malus yunnanensis, Viburnum betulifolium* |
|  | evergreen broadleaf tree | *Rhododendron polylepis, Abies fabri, Larix potaninii var. marcrocarpa, Picea likiangensis, Ilex pernyi* |
|  | deciduous broadleaf shrub | *Rubus pungens var. oldhamii, Smilax stans, Ribes glaciale, Meliosma cuneifolia* |
|  | evergreen broadleaf shrub | *Berberis potaninii* |
|  | liana | *Sabia yunnanensis subsp. Latifolia, Schisandra grandiflora* |
|  | forb | *Artemisia argyi, Thalictrum javanicum, Ophiopogon bodinieri, Rubia schumanniana, Valeriana officinalis, Brachypodium sylvaticum, Arundinaria faberi* |
|  | pteridophyte | *Lepisorus thunbergianus* |
| 2782 | deciduous broadleaf tree | *Acer flabellatum, Acer oliverianum, Acer laxiflorum, Betula utilis, Maddenia wilsonii, Ilex macrocarpa* |
|  | needleleaf evergreen tree | *Picea likiangensis, Abies fabri, Tsuga chinensis* |
|  | deciduous broadleaf small tree | *Enkianthus chinensis, Viburnum nervosum, Viburnum cylindricum, Malus prattii, Euonymus semenovii, Litsea chunii, Sorbus rufopilosa, Rubus delavayi, Ribes glaciale, Cerasus trichostoma* |
|  | evergreen broadleaf small tree | *Rhododendron calophytum, Rhododendron polylepis* |
|  | deciduous broadleaf shrub | *Hydrangea robusta* |
|  | evergreen broadleaf shrub | *Ilex yunnanensis* |
|  | liana | *Clematoclethra scandens subsp. actinidioides, Rubia cordifolia, Clematis kweichowensis, Clematis montana* |
|  | forb | *Pilea martini, Oxalis griffithii, Ligularia dentata, Arisaema elephas, Beesia calthifolia, Arundinaria faberi* |
| 2950 | deciduous broadleaf tree | *Acer laxiflorum, Betula utilis, Sorbus rufopilosa, Sorbus pallescens, Tilia chinensis var. intonsa* |
|  | deciduous broadleaf small tree | *Cerasus trichostoma, Litsea chunii, Viburnum betulifolium, Cotoneaster bullatus, Dipelta yunnanensis* |
|  | evergreen broadleaf tree | *Tsuga chinensis, Abies fabri, Rhododendron pachytrichum, Larix potaninii var. marcrocarpa* |
|  | evergreen broadleaf small tree | *Rhododendron decorum* |
|  | deciduous broadleaf shrub | *Rubus fockeanus, Lonicera tangutica, Rosa moyesii, Salix cathayana, Buddleja davidii* |
|  | evergreen broadleaf shrub | *Berberis potaninii* |
|  | forb | *Polygonum aviculare, Parasenecio palmatisectus, Artemisia argyi, Gentiana robusta, Arundinaria faberi* |
|  | pteridophyte | *Athyrium niponicum* |
| 2966 | deciduous broadleaf tree | *Betula utilis, Padus buergeriana, Sorbus pallescens, Acer laxiflorum, Populus kangdingensis* |
|  | deciduous broadleaf small tree | *Viburnum betulifolium, Sorbus rufopilosa, Litsea chunii, Viburnum nervosum, Cerasus trichostoma, Sorbaria arborea, Cotoneaster bullatus* |
|  | evergreen broadleaf tree | *Abies forrestii, Larix potaninii var. australis* |
|  | deciduous broadleaf shrub | *Berberis tischleri, Rosa moyesii, Lonicera lanceolata, Lonicera tangutica* |
|  | evergreen broadleaf shrub | *Berberis potaninii, Daphne tangutica* |
|  | liana | *Schisandra grandiflora* |
|  | forb | *Impatiens tortisepala, Parasenecio palmatisectus, Parasenecio roborowskii, Ligusticum daucoides, Brachypodium sylvaticum, Heracleum hemsleyanum* |
|  | pteridophyte | *Athyrium niponicum, Lepisorus thunbergianus* |
| 2993 | deciduous broadleaf tree | *Betula utilis, Acer kungshanense, Acer laxiflorum, Maddenia wilsonii* |
|  | needleleaf evergreen tree | *Abies fabri* |
|  | deciduous broadleaf small tree | *Sorbus prattii, Viburnum betulifolium, Viburnum nervosum, Philadelphus purpurascens, Cotoneaster bullatus, Rubus pungens var. oldhamii, Cerasus trichostoma, Euonymus frigidus, Padus buergeriana* |
|  | evergreen broadleaf small tree | *Rhododendron decorum, Rhododendron calophytum* |
|  | deciduous broadleaf shrub | *Ribes longiracemosum, Lonicera tangutica, Arundinaria faberi, Ribes glaciale, Rosa omeiensis* |
|  | evergreen broadleaf shrub | *Berberis aemulans* |
|  | liana | *Clematis montana, Smilax stans, Clematoclethra scandens subsp. actinidioides* |
|  | forb | *Streptopus obtusatus, Salvia smithii, Arisaema elephas, Berneuxia thibetica, Galium hoffmeisteri* |
| 3251 | evergreen needleleaf tree | *Abies fabri* |
|  | deciduous broadleaf small tree | *Acer stachyophyllum* |
|  | evergreen broadleaf small tree | *Rhododendron calophytum, Rhododendron lutescens, Rhododendron pachytrichum* |
|  | deciduous broadleaf shrub | *Sorbus rufopilosa, Acer flabellatum, Rubus pungens, Lonicera tangutica, Lonicera nigra, Euonymus semenovii, Rosa omeiensis, Philadelphus purpurascens, Ribes glaciale* |
|  | liana | *Clematis montana* |
|  | forb | *Maianthemum henryi, Arisaema elephas, Galium innocuum, Parasenecio deltophyllus, Carex capilliformis* |
| 3290 | deciduous broadleaf tree | *Acer pictum subsp. mono, Malus rockii, Populus davidiana, Cerasus pleiocerasus, Sorbus thibetica, Salix wallichiana* |
|  | evergreen needleleaf tree | *Picea likiangensis var. hirtella* |
|  | evergreen broadleaf tree | *Quercus guyavifolia* |
|  | deciduous broadleaf shrub | *Rosa soulieana, Zanthoxylum undulatifolium, Rhamnus maximovicziana, Ribes alpestre, Cotoneaster tenuipes* |
|  | liana | *Berchemia yunnanensis* |
|  | forb | *Chrysanthemum glabriusculum, Thalictrum atriplex, Anemone tomentosa, Salvia brevilabra, Elsholtzia ciliata* |
|  | pteridophyte | *Onychium contiguum* |
| 3500 | deciduous broadleaf tree | *Hippophae rhamnoides, Cerasus serrula* |
|  | deciduous broadleaf small tree | *Malus transitoria* |
|  | evergreen needleleaf tree | *Picea brachytyla, Juniperus pingii* |
|  | evergreen broadleaf tree | *Quercus guyavifolia* |
|  | deciduous broadleaf shrub | *Caragana franchetiana, Berberis approximata, Ribes alpestre, Spiraea schneideriana* |
|  | evergreen broadleaf shrub | *Rhododendron intricatum* |
|  | evergreen needleleaf shrub | *Juniperus pingii var. wilsonii* |
|  | forb | *Halenia elliptica, Stellera chamaejasme, Thalictrum cultratum, Polygonatum cirrhifolium, Salvia prattii, Carex cardiolepis* |
| 3780 | deciduous broadleaf tree | *Hippophae rhamnoides* |
|  | deciduous needleleaf tree | *Larix potaninii var. marcrocarpa* |
|  | evergreen needleleaf tree | *Picea likiangensis var. hirtella, Pinus yunnanensis* |
|  | evergreen broadleaf small tree | *Quercus guyavifolia, Rhododendron intricatum* |
|  | deciduous broadleaf shrub | *Potentilla fruticosa, Berberis approximata, Caragana erinacea* |
|  | evergreen needleleaf shrub | *Juniperus pingii var. wilsonii* |
|  | liana | *Clematis tangutica* |
|  | forb | *Stellera chamaejasme, Spenceria ramalana, Anaphalis flavescens, Potentilla saundersiana, Anemone tomentosa, Deyeuxia scabrescens* |
| 3794 | deciduous broadleaf shrub | *Sibiraea angustata, Salix wuxuhaiensis, Rhododendron intricatum, Caragana erinacea, Lonicera rupicola, Potentilla fruticosa var. arbuscula* |
|  | evergreen broadleaf shrub | *Berberis dictyoneura, Quercus guyavifolia* |
|  | evergreen needleleaf shrub | *Juniperus pingii var. wilsonii* |
|  | forb | *Sibbaldia cuneata, Potentilla anserina, Stellera chamaejasme, Anaphalis aureopunctata, Anemone tomentosa, Carex cardiolepis* |
| 3943 | deciduous broadleaf shrub | *Berberis dictyophylla, Salix sclerophylla, Salix spodiophylla, Lonicera ligustrina, Lonicera rupicola var. syringantha, Spiraea schneideriana, Potentilla fruticosa, Sorbus rehderiana, Ribes takare* |
|  | evergreen broadleaf shrub | *Rhododendron phaeochrysum, Rhododendron intricatum* |
|  | forb | *Rheum nobile, Gentiana trichotoma, Polygonum macrophyllum, Sedum chauveaudii, Ligularia duciformis, Angelica sinensis* |
| 4081 | deciduous broadleaf shrub | *Sorbus rehderiana, Salix sclerophylla, Potentilla fruticosa, Ribes takare, Spiraea schneideriana, Anaphalis souliei* |
|  | evergreen broadleaf shrub | *Rhododendron intricatum, Rhododendron phaeochrysum, Lonicera ligustrina* |
|  | evergreen needleleaf shrub | *Picea likiangensis* |
|  | forb | *Ligularia pleurocaulis, Polygonum macrophyllum, Gentiana trichotoma, Pyrethrum tatsienense, Potentilla stenophylla var. emergens, Poa attenuata, Carex cardiolepis* |
| 4361 | deciduous broadleaf shrub | *Salix flabellaris, Lonicera hispida* |
|  | evergreen broadleaf shrub | *Rhododendron intricatum, Rhododendron telmateium, Rhododendron phaeochrysum* |
|  | forb | *Polygonum macrophyllum, Saussurea przewalskii, Hedysarum vicioides, Gentiana trichotoma, Potentilla stenophylla var. emergens, Trisetum spicatum, Carex cardiolepis* |
|  | pteridophyte | *Polystichum gongboense* |

**Fig. S1. The locations of weather stations used to derive estimates of the climate variables at each sampled site.** The red triangles are the weather stations and dots are the sampling sites. The background colour represents the elevation gradient which has the same scale in Fig. 1 in the main text.

**
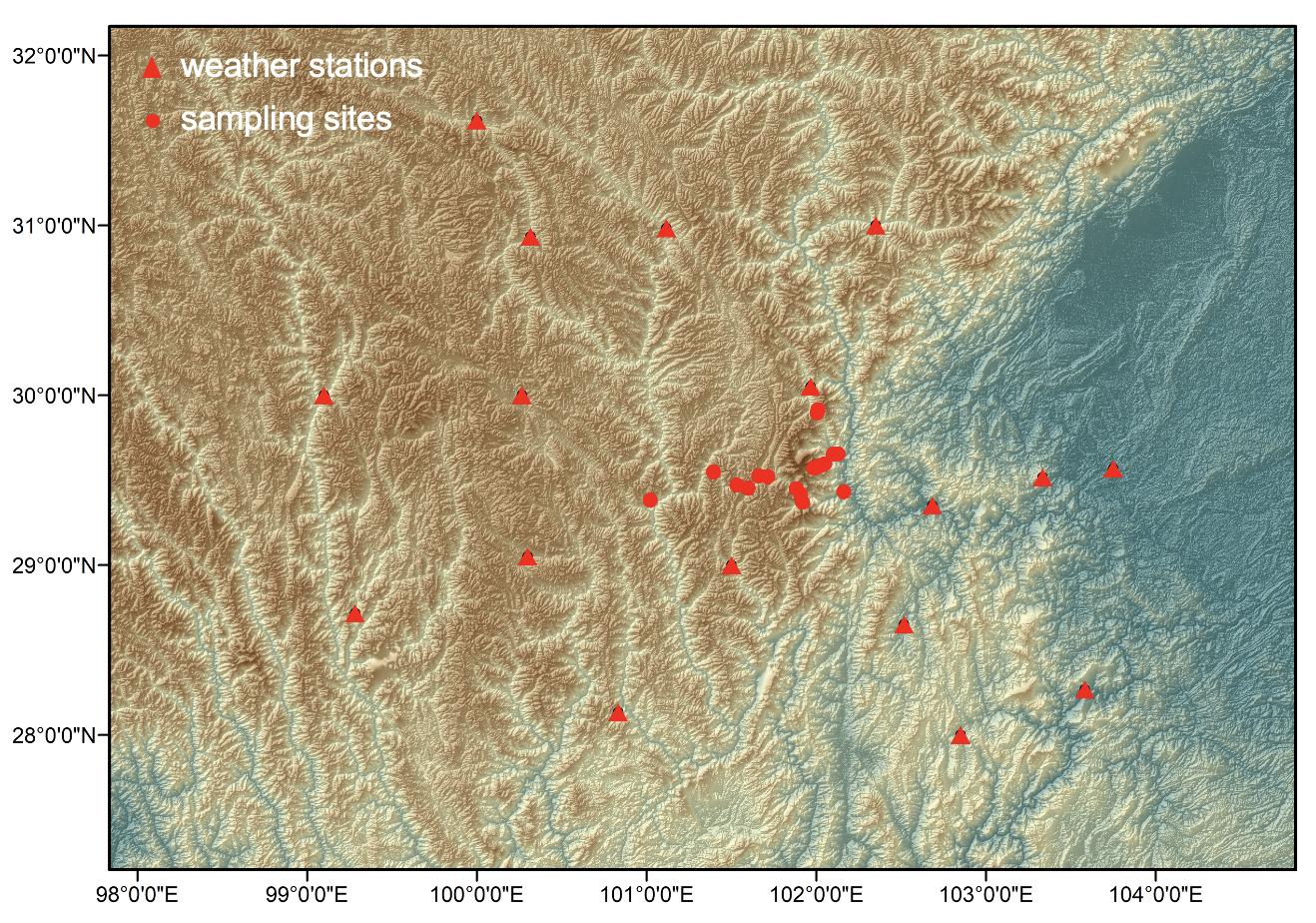
**

**Fig. S2. The comparisons of interpolated and in-situ climate data at five sites.** The red dots are the interpolated climate data and blue dots are the in-situ climate data collected from flux tower or nearby meteorological stations. The bioclimatic variables are daytime temperature in July (*T*_dJ_), mean temperature during the growing season, defined as days above a baseline of 0 ˚C (*T*_g_), mean vapor pressure deficit (*D*_0_), mean photosynthetically active radiation (*R*_0_), and a moisture index (α_p_) defined as the ratio of annual actual evapotranspiration to annual potential evapotranspiration.


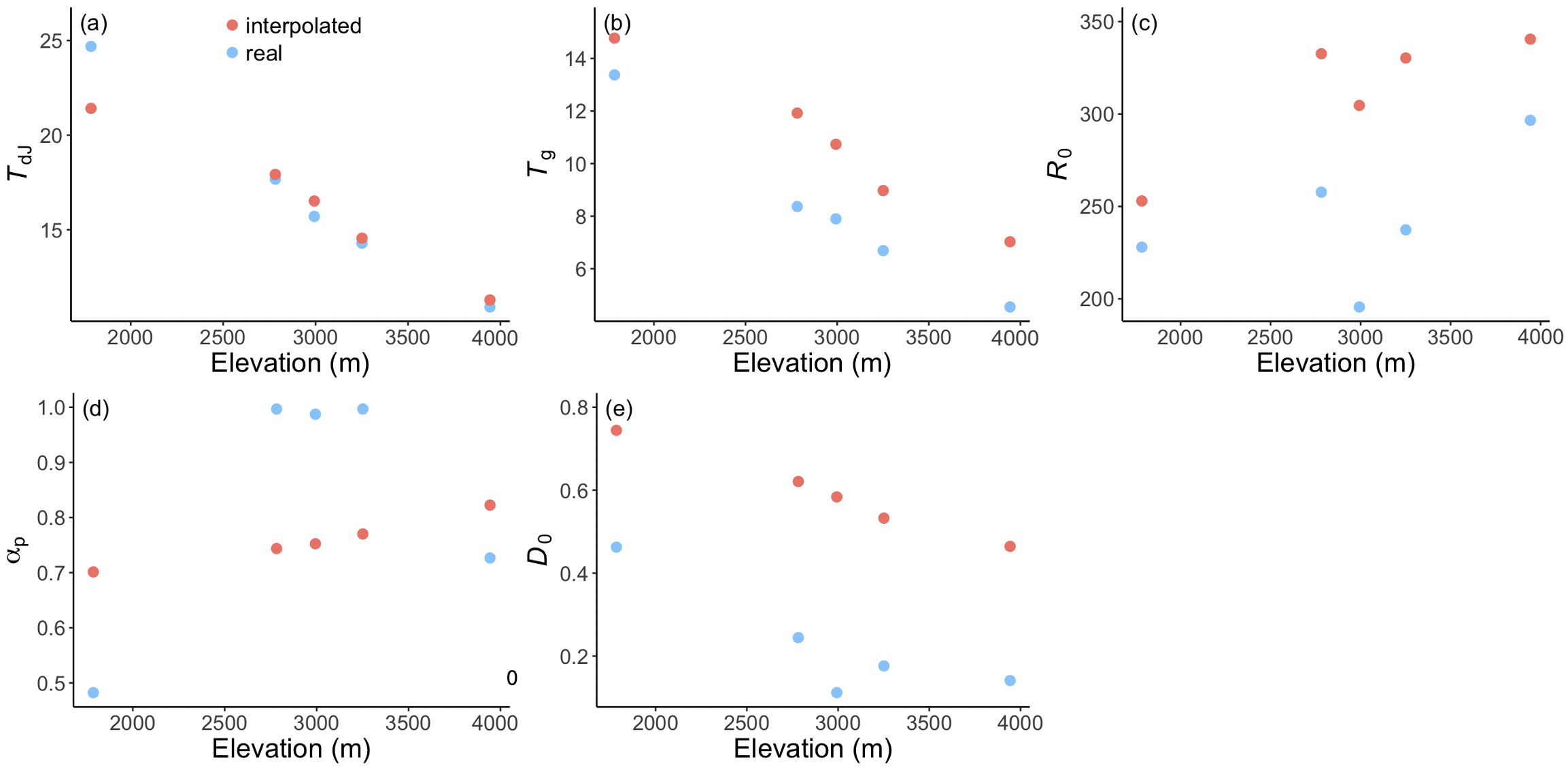


**Fig. S3. The coefficient of variation (CV) of traits at each sampled site.** The traits are leaf mass per area (*M*_a_), leaf nitrogen content per unit area (*N*_area_); the maximum capacity of carboxylation standardized to 25 ˚C (*V*_cmax25_) and the ratio of leaf-internal to ambient CO_2_ partial pressure (χ). The red dashed line is the CV of traits across sites.


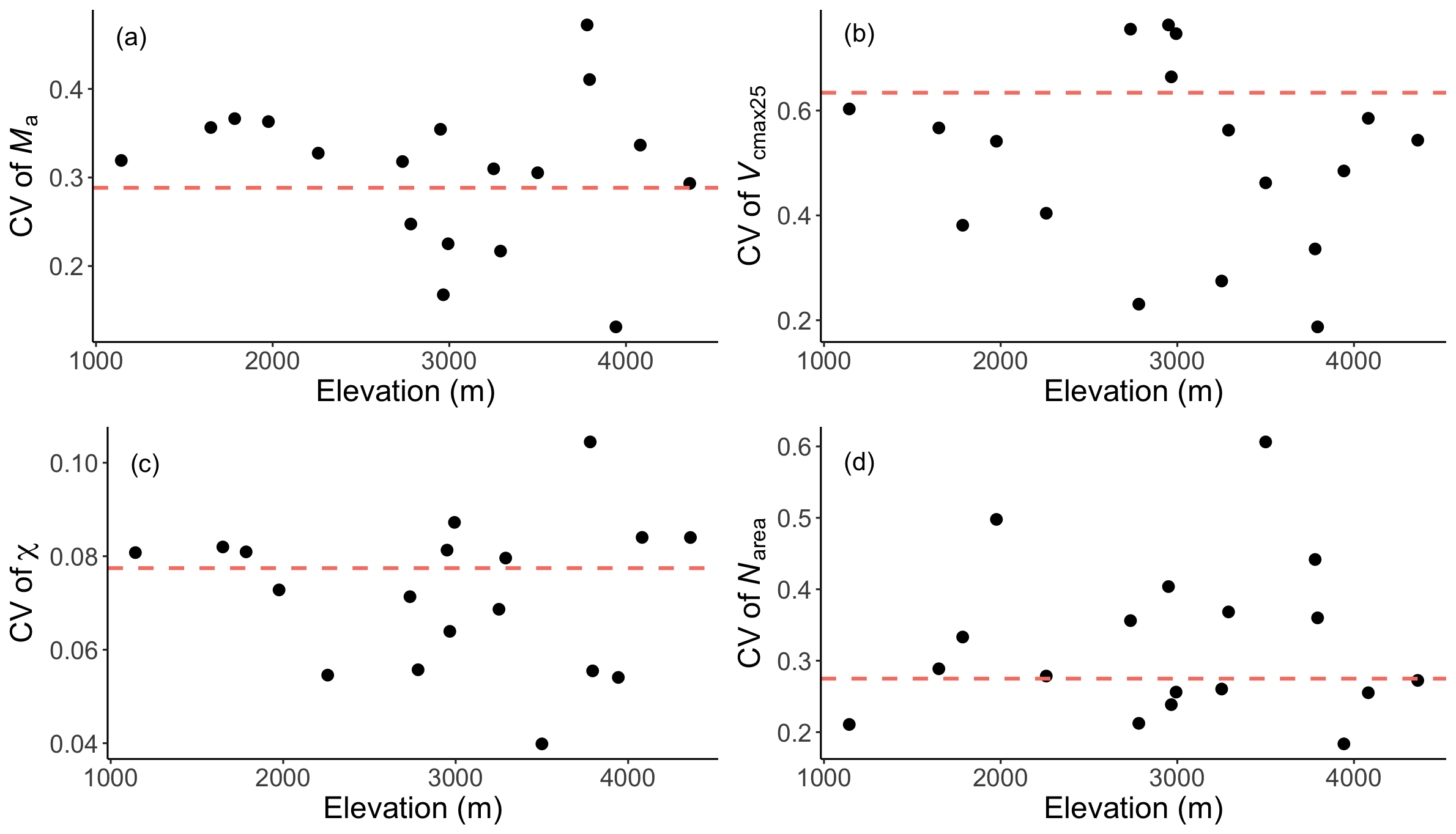


**Fig. S4. Site-mean values of the maximum capacity of carboxylation standardized to 25 ˚C (*V*_cmax25_) and the ratio of leaf-internal to ambient CO_2_ partial pressure (χ) for evergreen species.** Observations are site-mean values of evergreen species. The solid line is the 1:1 line.

**
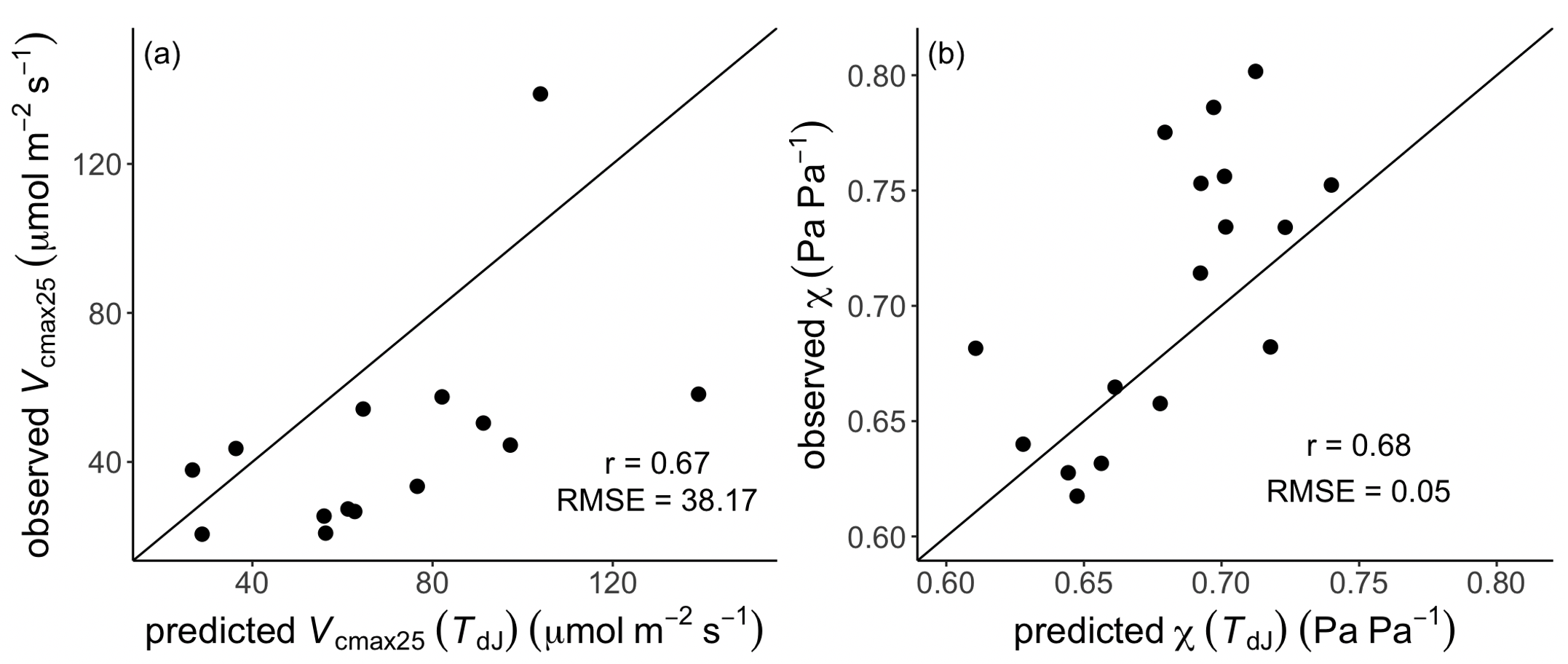
**

**Fig. S5. The observed and predicted values of traits along the altitudinal gradient.** The traits are leaf mass per area (*M*_a_), leaf nitrogen content per unit area (*N*_area_); the maximum capacity of carboxylation standardized to 25 ˚C (*V*_cmax25_) and the ratio of leaf-internal to ambient CO_2_ partial pressure (χ). Observed trait values are shown in grey dots. In panel (a), the red and blue dots show the mean predicted *M*_a_ using mean temperature during the growing season (*T*_g_) with regional-calibrated coefficients in Eq. (15) and using daily temperature in July (*T*_dJ_), respectively. In panel (b) and (c), red dots show the mean predicted *V*_cmax25_ and χ using *T*_g_. In panel (d), predicted *N*_area_ using *N*_structure_ and *N*_rubisco_ following Dong et al. (2017) are shown with red dots, the blue dots show the mean *N*_area_ predicted from predicted *T*_g_-driven *M*_a_ and *T*_dJ_-driven *V*_cmax25_. Error bars in panel (a) and (c) are the square root of uncertainty caused by parameters, in panel (b) by parameters and observed χ, in panel (d) by parameters and observed *M*_a_ and *V*_cmax25_.


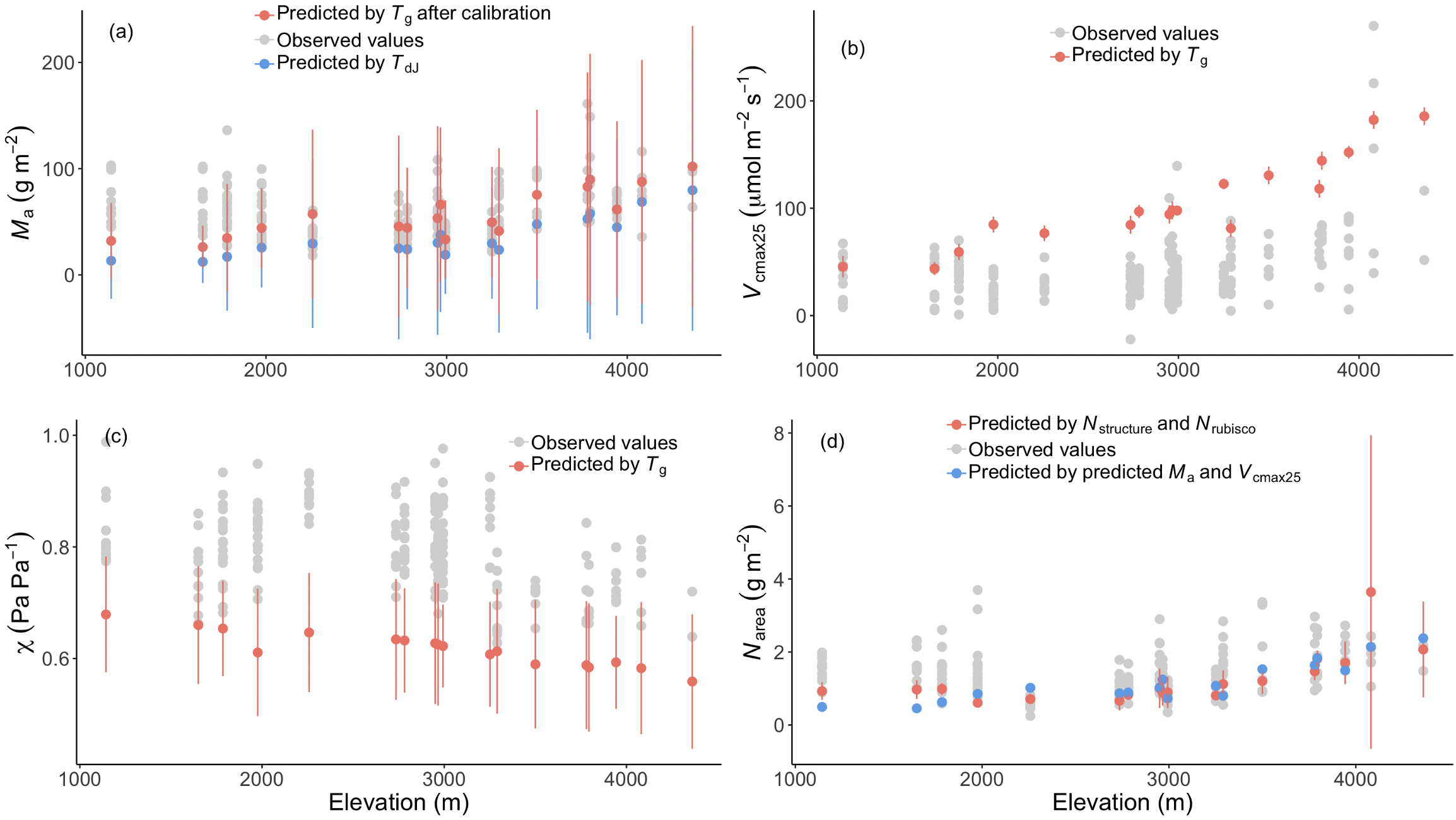


**Fig. S6. Site-mean values of traits for deciduous species.** The traits are leaf mass per area (*M*_a_), leaf nitrogen content per unit area (*N*_area_); the maximum capacity of carboxylation standardized to 25 ˚C (*V*_cmax25_) and the ratio of leaf-internal to ambient CO_2_ partial pressure (χ). Observations are site-mean values of deciduous species. In panel (a), the red and blue dots show the mean predicted *M*_a_ using mean temperature during the growing season (*T*_g_) with regional-calibrated coefficients in Eq. (15) and using daily temperature in July (*T*_dJ_), respectively. In panel (b) and (c), the red dots show the mean predicted *V*_cmax25_ and χ using *T*_g_. In panel (d), predicted *N*_area_ using *N*_structure_ and *N*_rubisco_ following Dong et al. (2017) are shown with red dots, the blue dots show the mean *N*_area_ predicted from predicted *T*_g_-driven *M*_a_ and *T*_dJ_-driven *V*_cmax25_. The solid line is the 1:1 line.


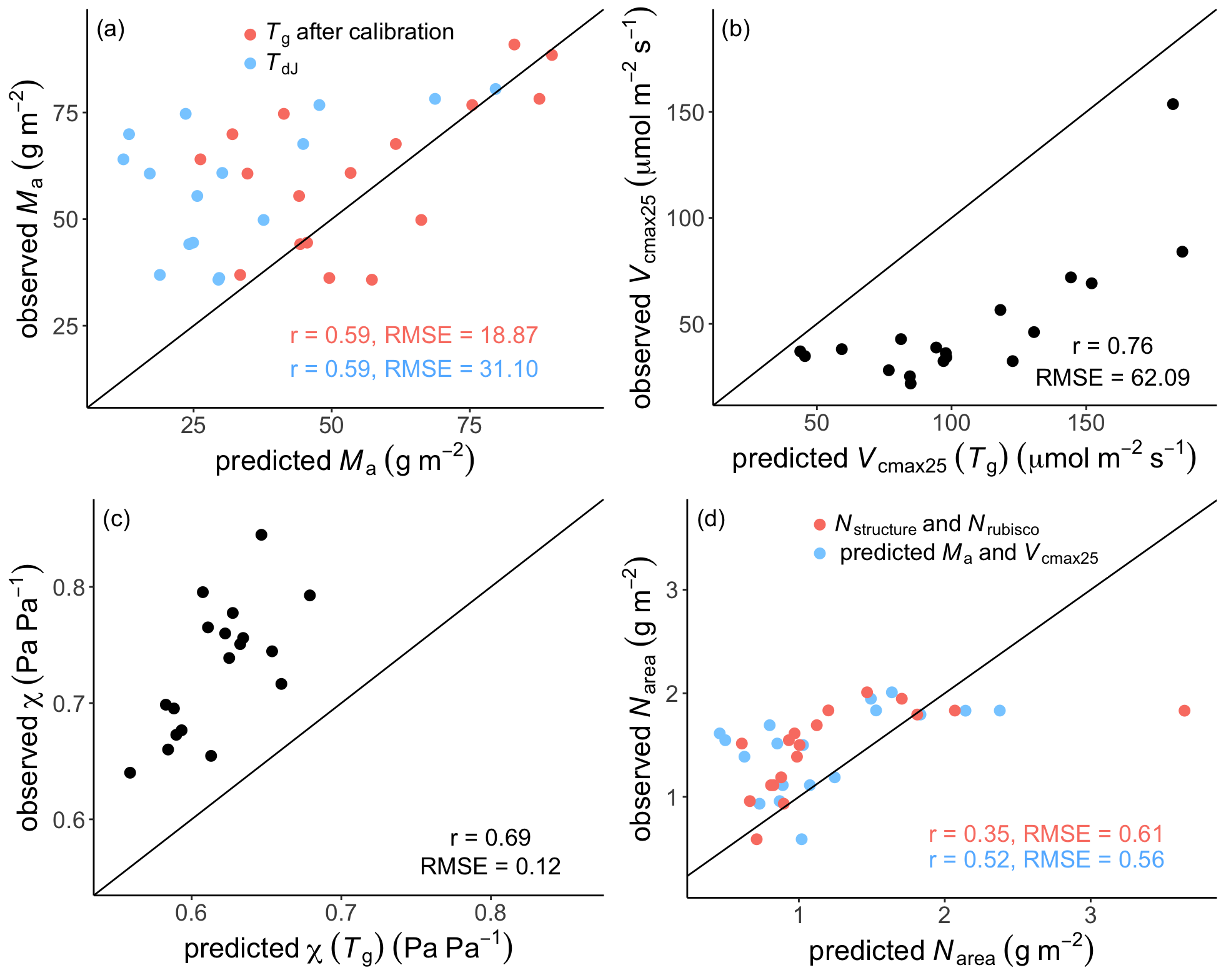


**Fig. S7. The impact of parameter uncertainty on the prediction of traits.** Site at 1785 m was selected as a case study. Blue circles are predicted values at 1785 m and orange circles are mean observed values. Error bar is accumulated uncertainty by successive parameters on y-axis from the bottom to the top. *M*_a_ is predicted leaf mass per area using *T*_g_, *V*_cmax25_ is predicted maximum capacity of carboxylation standardized to 25 ˚C using *T*_dJ_, χ is predicted ratio of internal to ambient CO_2_ partial pressure using *T*_dJ_, and *N*_area_ is predicted leaf mass per area by observed *M*_a_ and *V*_cmax25_ directly. *c* is the unit cost of maintaining electron transport capacity, *K*_c_ (Pa) is Michaelis-Menten coefficient for carboxylation at 25 ˚C, Δ*H*_K_C_ (J mol^-1^) is the activation energy of carboxylation, Δ*H*_Γ*_ (J mol^-1^) is the activation energy of Γ^*^, Γ^*^_25_ (Pa) is photorespiratory compensation point at 25 ˚C, Δ*H*_K_O_ (J mol^-1^) is the activation energy of oxygenation. β is the ratio of the unit costs of maintaining carboxylation and transpiration capacities.


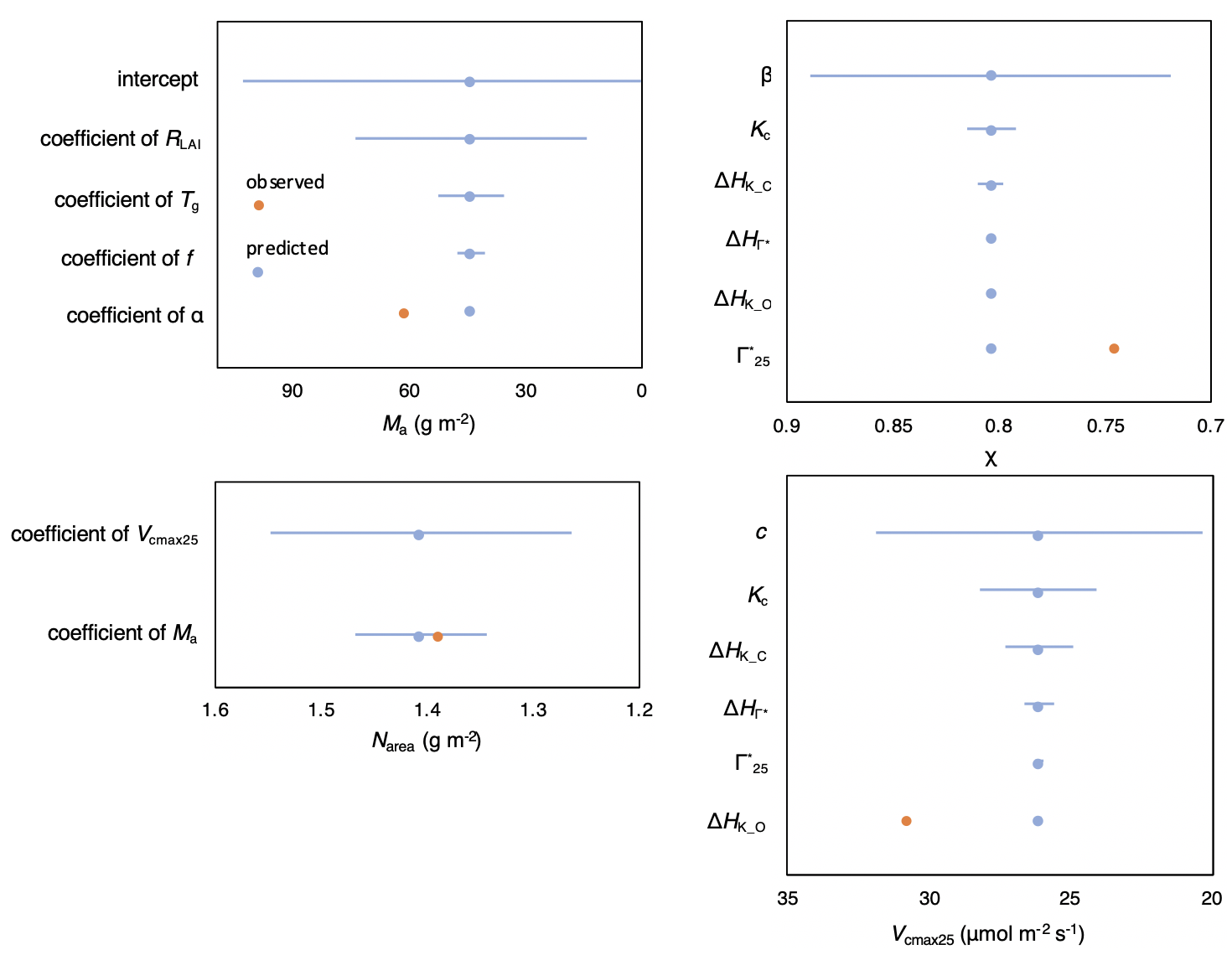


**Fig. S8. The distribution of observed leaf nitrogen content per unit area (*N*_area_) values in the space of leaf mass per area (*M*_a_) and the maximum capacity of carboxylation standardized to 25** **˚C (*V*_cmax25_).** Trait values are indicated by the colour scale.

**
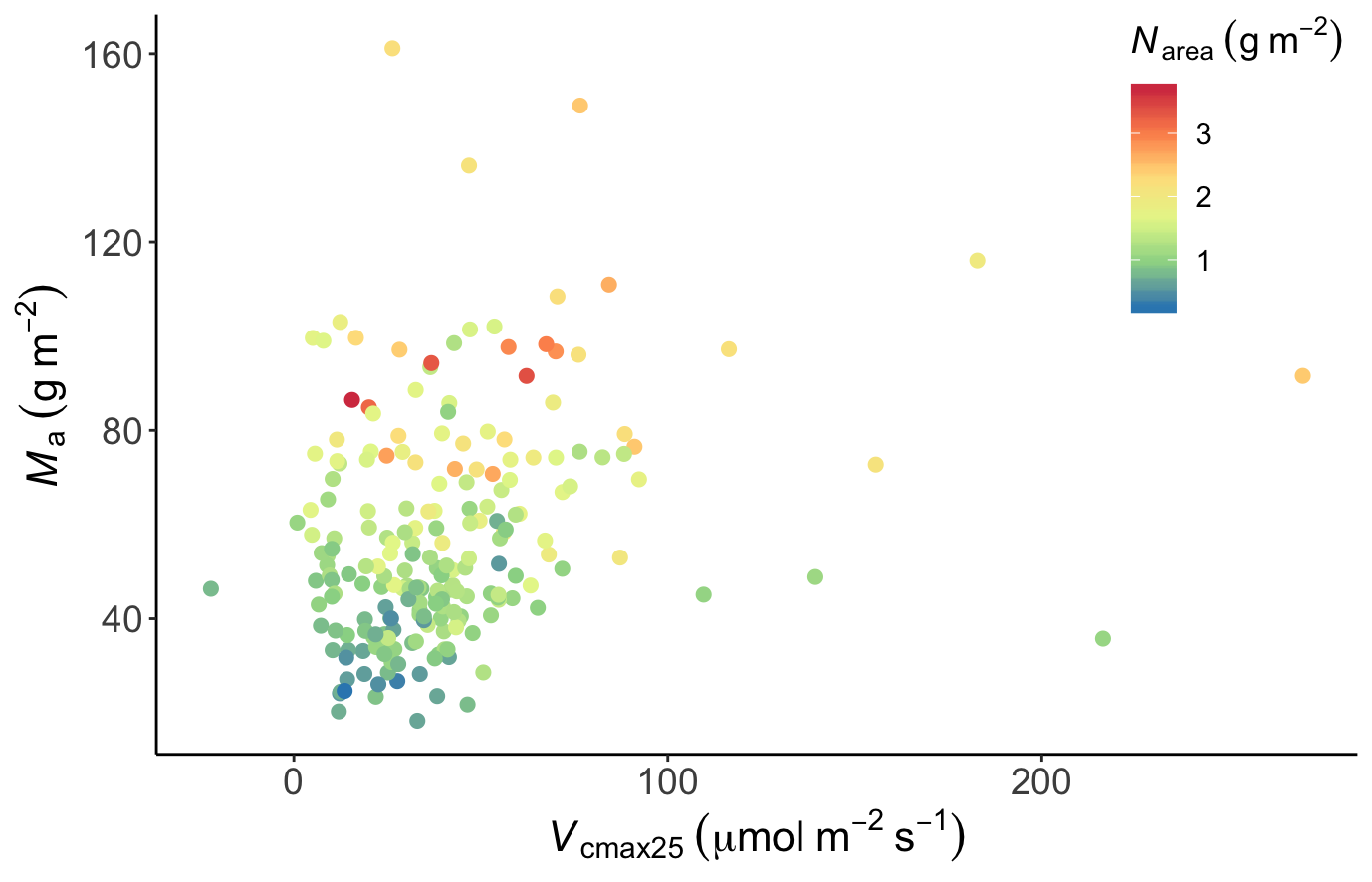
**

**Fig. S9. The relationships between residual of predictions and hydraulic traits and α_p_.** Only the significant relationships were represented. *T*_g_ means predicted traits using mean temperature during growing season, *T*_dJ_ means predicted traits using daytime temperature of July. α_p_ is the ratio of annual actual evapotranspiration to annual potential evapotranspiration.


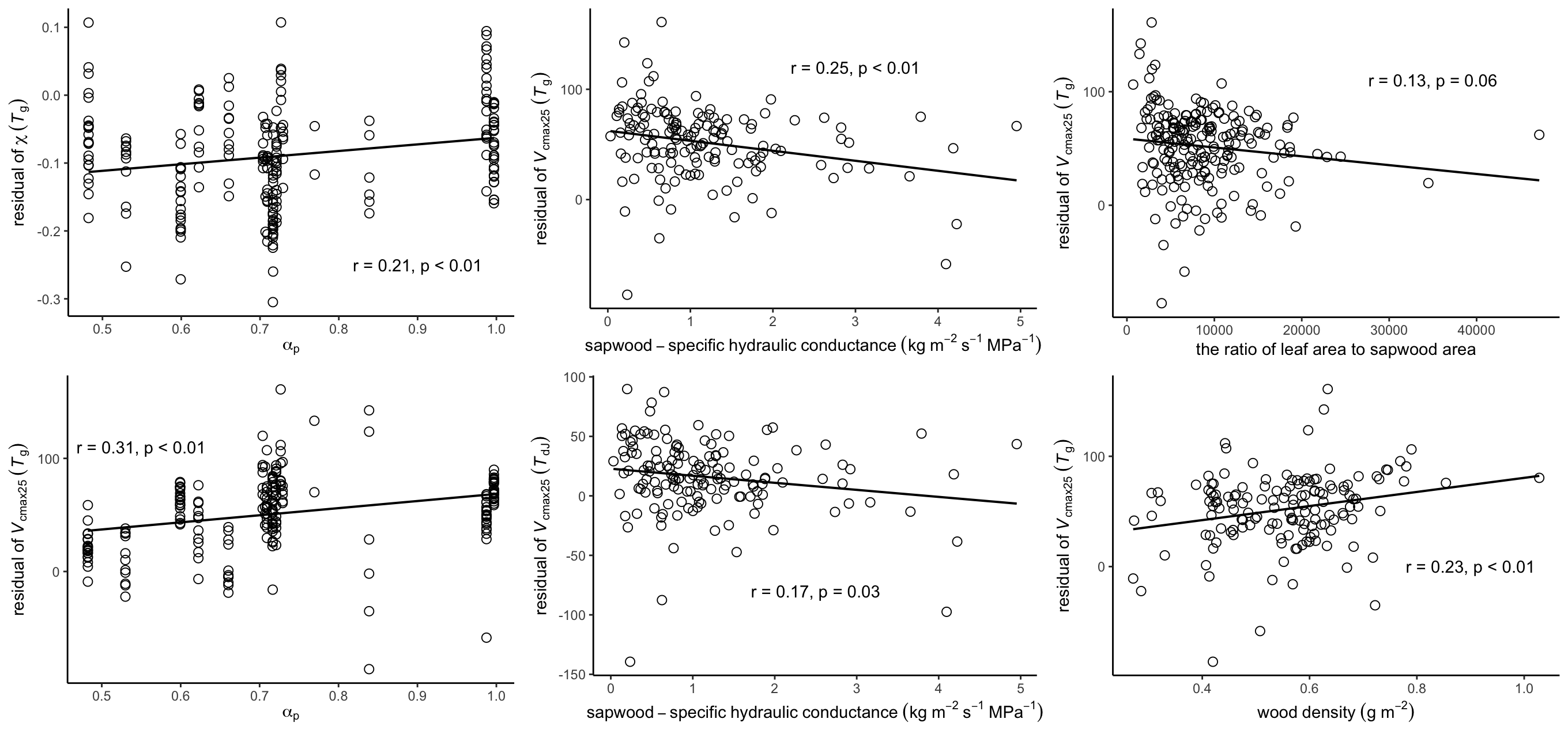

Supplement: Supplementary_tpab003 [file supplementary_tpab003.docx]
